# Fluid absorption by skin tissue during intradermal injections through hollow microneedles

**DOI:** 10.1038/s41598-018-32026-9

**Published:** 2018-09-13

**Authors:** Pranav Shrestha, Boris Stoeber

**Affiliations:** 1 Department of Mechanical Engineering, The University of British Columbia, 2054-6250 Applied Science Lane, Vancouver, British Columbia V6T 1Z4 Canada; 2 Department of Electrical and Computer Engineering, The University of British Columbia, 2332 Main Mall, Vancouver, British Columbia V6T 1Z4 Canada

## Abstract

Hollow microneedles are an emerging technology for delivering drugs and therapeutics, such as vaccines and insulin, into the skin. Although the benefits of intradermal drug delivery have been known for decades, our understanding of fluid absorption by skin tissue has been limited due to the difficulties in imaging a highly scattering biological material such as skin. Here, we report the first real-time imaging of skin tissue at the microscale during intradermal injections through hollow microneedles, using optical coherence tomography. We show that skin tissue behaves like a deformable porous medium and absorbs fluid by locally expanding rather than rupturing to form a single fluid filled cavity. We measure the strain distribution in a cross section of the tissue to quantify local tissue deformation, and find that the amount of volumetric expansion of the tissue corresponds closely to the volume of fluid injected. Mechanically restricting tissue expansion limits fluid absorption into the tissue. Our experimental findings can provide insights to optimize the delivery of drugs into skin for different therapeutic applications, and to better model fluid flow into biological tissue.

## Introduction

Skin, the largest organ of the human body, provides a physical barrier against chemicals and micro-organisms and generates innate and adaptive immune responses. Due to the skin’s immune response and access to capillary and lymphatic systems, the three layers of the skin are potential sites for drug delivery – transdermal, intradermal and subcutaneous drug delivery into the epidermis, dermis and hypodermis, respectively^1,2^. The advantages of intradermal injection of drugs have been known for decades. Some vaccines generate equivalent immune response with a significantly reduced dose for intradermal injections than for deeper injections, due to epidermal Langerhans and dermal dendritic cells^2^. In addition, drugs are not prematurely metabolized by the gastro-intestinal tract and liver, as for oral drug administration, thus eliminating the first-pass effect^3^. Despite the advantages, widespread use of intradermal drug delivery has been historically limited due to the drawbacks of the standard intradermal injection technique, the Mantoux method, such as the difficulty in performing the technique requiring highly trained medical personnel, the poor consistency of the injected volume, the invasive nature of hypodermic needle injections, the potential for needle-stick injuries, the risk of transmitting blood-borne pathogens through inappropriate needle re-use, and perhaps most importantly the risk of accidental subcutaneous injections^2,4^. If a patient receives a reduced dose of a vaccine, to take advantage of the dose sparing effect in intradermal delivery, and the injection ends up deeper in the skin, the patient will not be immunized. However, recent developments of novel devices, for easier and more reliable intradermal injections, such as microneedles have renewed interest in intradermal drug delivery^2,5–7^.

Hollow microneedles are sub-millimeter devices that penetrate the outermost layer of the epidermis, the stratum corneum (SC), to inject drugs, such as biotherapeutics (e.g. insulin) and vaccines (e.g. influenza vaccine), into the dermis or epidermis. In addition to eliminating most of the drawbacks of the standard Mantoux technique, hollow microneedles can improve patient compliance, and potentially permit self-administration^8,9^. Studies have shown that the primary resistance to fluid flow during intradermal injections is caused by the skin, rather than the microneedle, and that partial retraction of the microneedle decreases the dermal resistance and increases flow^10^. Previous experiments^10–13^ have been limited to studying the effect of injection parameters on flow – the fluid flow-rate into skin increased by increasing the fluid injection pressure, the microneedle insertion depth, the microneedle retraction distance, and using a beveled microneedle tip. Currently, however, our understanding of how fluid is absorbed by the skin during intradermal injections is limited. This lack of understanding, which is essential for controlling effective delivery of fluid into the skin, could be attributed to challenges associated with visualizing intradermal injections because skin tissue is a highly optically scattering biological material^14^.

Biological tissue can be considered as a porous medium for modelling fluid flow^15^, and soft tissue, such as skin tissue, can be considered as a deformable porous medium: the pressure associated with the injected fluid can deform the soft porous matrix as fluid flows through its pores. Skin tissue has also been considered analogous to a sponge filled with fluid by describing its mechanical behaviour using mixture theory^16^. When the sponge is compressed, fluid flows out of the sponge (with the viscosity of the fluid resisting flow) and the size of its pores decreases (increasing the resistance to fluid flow) - resulting in a non-linear time-dependent behaviour. The current literature on simulations and mathematical models of injections into biological tissue has varying results due to differences in model assumptions. Tissue has been modelled as a mechanically non-linear deformable porous medium^17^, where injection into tissue formed a fluid-filled spherical cavity and high cavity pressure caused fluid to flow into the neighbouring tissue. Fluid flow and tissue deformation were found to be coupled and any flow induced deformation of the material increased local tissue permeability, aiding fluid transport. Another model considered the injection of fluid into a layer of deformable porous medium from a point source^18^, with applications to subcutaneous injections, assuming linear poro-elasticity and a constant permeability of the medium. Simulations using these assumptions indicated a swelling of the porous medium (with no cavity formation) and a subsequent deformation of the free surface, resembling the raised papule (wheal or bleb) observed after successful intradermal injections. Other models have considered flow through skin as porous medium without deformations^19–21^, flow into a growing cavity in non-porous medium^22^, or a combination of spherical expansion in non-porous skin followed by spherical diffusion in porous skin^23^. Researchers have also used finite element modeling of microneedle insertion into skin to indirectly predict fluid flow, by assuming stresses developed in the tissue change local permeability^24^. For creating more accurate models, we require experimental observations of flow through tissue to validate model assumptions and to provide physical insights into the dynamic behavior of tissue during injections.

To visualize the dynamics of intradermal injections, an imaging system requires micron-level spatial resolution, an imaging depth greater than 1 mm in a highly scattering biological medium, and a temporal resolution high enough to capture tissue or fluid movement without significant motion artifacts. One such non-invasive biomedical imaging modality is optical coherence tomography (OCT), which produces two-dimensional (2D) cross-sectional images of internal tissue microstructures in real-time^25^. OCT has been widely used in ophthalmology^26^ and dermatology^27,28^, due to its ability to perform non-invasive “optical biopsy” on tissue with micron-level resolution up to depths of 1–2 mm. Previously, the use of OCT had been limited to imaging microneedle penetration into skin tissue^29,30^, or dissolution of polymeric microneedles^31^. Other reported imaging of tissue were mostly carried out post-injection or post-insertion using confocal microscopy^5,32–34^, ultrasound echography^12^, fluorescence microscopy^10,35^, histology^10,11,33,36,37^, x-ray computed tomography (micro-CT)^38,39^, or two-photon microscopy^40^. Fluorescence and confocal microscopy have adequate spatial and temporal resolutions to image injections, but have low imaging depths in skin (up to few hundred micrometers) that only allow visualizing the upper layers of skin after injections. Micro-CT and two-photon microscopy have better imaging depths, but are not suitable for real-time imaging of skin cross-sections. The only reported real-time visualization of injections into skin tissue was for deeper subcutaneous injections using high-speed X-ray imaging^41,42^. However, high-speed imaging techniques using X-ray or ultrasound do not have micron-level spatial resolution required for imaging intradermal injections.

**Figure 1:**
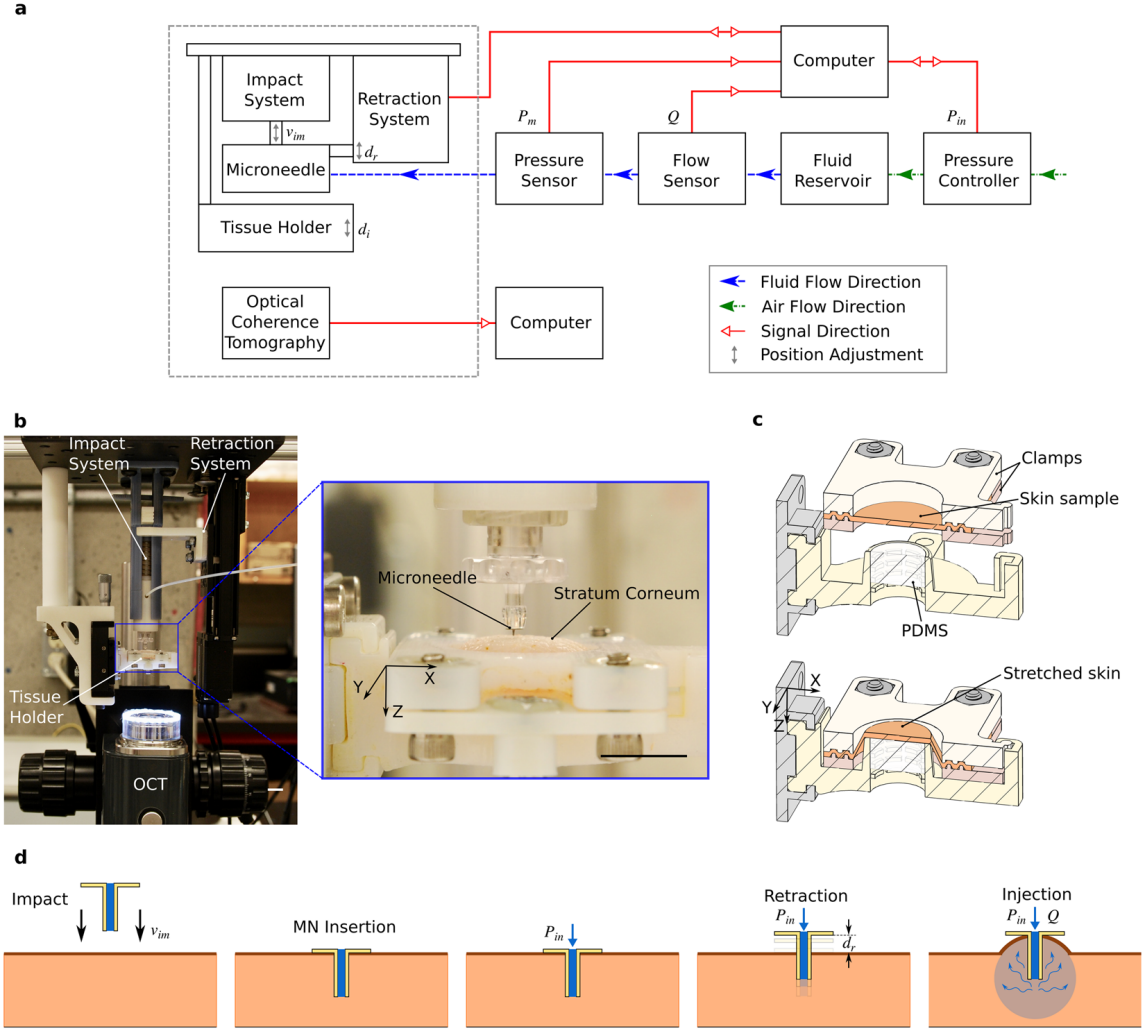
Experimental setup and injection procedure. (**a**) Simplified schematic of the experimental setup (not to scale) showing the major components and their interactions, with variables indicating injection parameters controlled and/or measured. (**b**) Picture of the injection system (dashed grey box in **a**), showing the impact system, retraction system, tissue holder and OCT. Detail (blue box) showing microneedle and excised porcine skin sample (stretched) in the tissue holder. The scale bars (white and black) are 10 mm. (**c)** Cross-sectional (mid-section) schematics of the tissue holder showing procedure to stretch skin to approximate *in-vivo* dimensions (Methods): clamping of the excised skin sample (top), and uniform stretching in radial directions (bottom) using transparent PDMS backing. (**d)** Injection procedure showing 5 steps: i) microneedle impacting the stretched skin (with velocity *v*_*im*_) to penetrate stratum corneum; ii) microneedle insertion; iii) application of input pressure (*P*_*in*_) with no initial flow due to high dermal resistance; iv) retraction of microneedle (by *d*_*r*_); v) successful injection of fluid with the formation of raised papule (characteristic of intradermal injections).

Here, we report the first use of OCT for real-time visualization of skin tissue deformation during intradermal injections through hollow microneedles. We present an experimental setup (Fig. 1a–c) to: control injection parameters such as fluid input pressure *P*_*in*_, microneedle impact velocity *v*_*im*_, insertion depth *d*_*i*_, and retraction distance *d*_*r*_; record fluid parameters such as pressure *P*_*m*_, and flow-rate *Q*; and visualize tissue microstructure using OCT. For all experiments, we used a hollow microneedle (height 700 μm; lumen diameter 100 μm; Fig. 1b) to inject water into excised porcine skin tissue. We first impacted the microneedle onto a stretched skin sample with a velocity *v*_*im*_ of approximately 2 m/s, to pierce the stratum corneum (Fig. 1d,i). When we applied an input pressure *P*_*in*_ = 100 kPa with the microneedle inserted, water was not injected into the skin (Fig. 1d,iii). Some researchers have attributed this inability to inject fluid initially to either the compression of dermal tissue around the microneedle or plugging of the microneedle lumen by dermal tissue during microneedle insertion^10^, which had motivated retracting the microneedles partially to relieve the tissue compression and/or remove cored tissue. The partial retraction of microneedles had been previously reported to increase the injection flow-rate up to 11.6-fold^11^. For our experiments, we found that retracting the microneedle by *d*_*r*_ = 0.3 mm consistently reduced the dermal resistance and triggered the onset of fluid flow (with *P*_*in*_ = 100 kPa). We observed the skin tissue during this injection using OCT.

## Results and Discussion

### Microinjection and microinfusion

**Figure 2:**
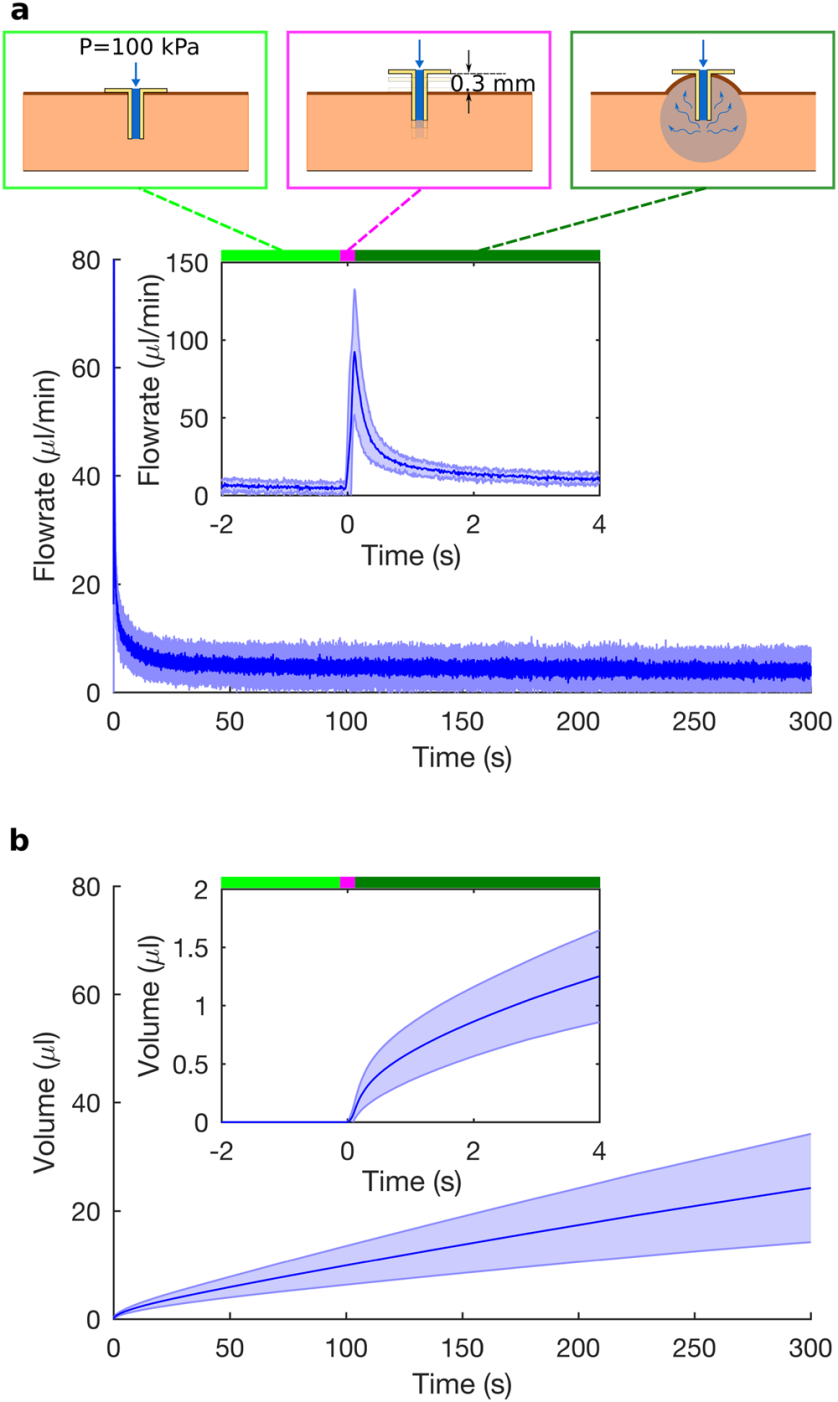
Two modes of flow – microinjection and microinfusion. (**a**) Flow-rate of water into excised porcine skin sample, showing the mean (solid blue line) and the standard deviation (blue shading) for five experiments. Water is initially not injected into the skin, even with an input pressure of 100 kPa (light green bar); the microneedle is retracted by 0.3 mm (magenta bar); and water flows into the skin (dark green bar; t = 0 s marking the onset of fluid flow during retraction). When the MN is retracted, the flow-rate increases rapidly (microinjection; inset after 0 s) and decays to a non-zero steady state value (micro-infusion; after approximately 10 s). (**b**) Volume of water injected into the skin, calculated by integrating the flow-rate over time, shows rapid fluid absorption by tissue during microinjection (inset) followed by slower fluid absorption during microinfusion.

After the onset of fluid flow due to microneedle (MN) retraction, we identified two modes of flow into tissue, defined here as: *microinjection* (region of high transient fluid flow-rate; Fig. 2a inset after 0 s), and *microinfusion* (region of steady-state flow-rate, lower than that in the transient case; Fig. 2a after ∼10 s). The sampling rate of the flow sensor was high enough (100 Hz) to capture the transient changes in fluid flow to enable identification of the two modes. The details of the transient flow-rate immediately following retraction had not been reported earlier either due to lower sampling rates^11^ or due to the use of a syringe pump to maintain a constant flow-rate^12^, instead of a constant pressure as in our experiments.

By integrating the flow-rate with respect to time, after the microneedle retraction (at t = 0 s), we calculated the total volume of water injected into the tissue as a function of time (Fig. 2b). The injected volume over time provided a measure of fluid absorption by the skin tissue at different stages of the injection. The tissue absorbed water more rapidly initially during microinjection than during microinfusion, as indicated by the steep slope of the volume-vs.-time curve initially followed by a much more gentle slope (Fig. 2b). As elaborated later, the different rates of fluid absorption, during microinjection and microinfusion, can be related to variations in rates of tissue expansion derived from OCT images.

### Real-time imaging of tissue microstructure using OCT

**Figure 3:**
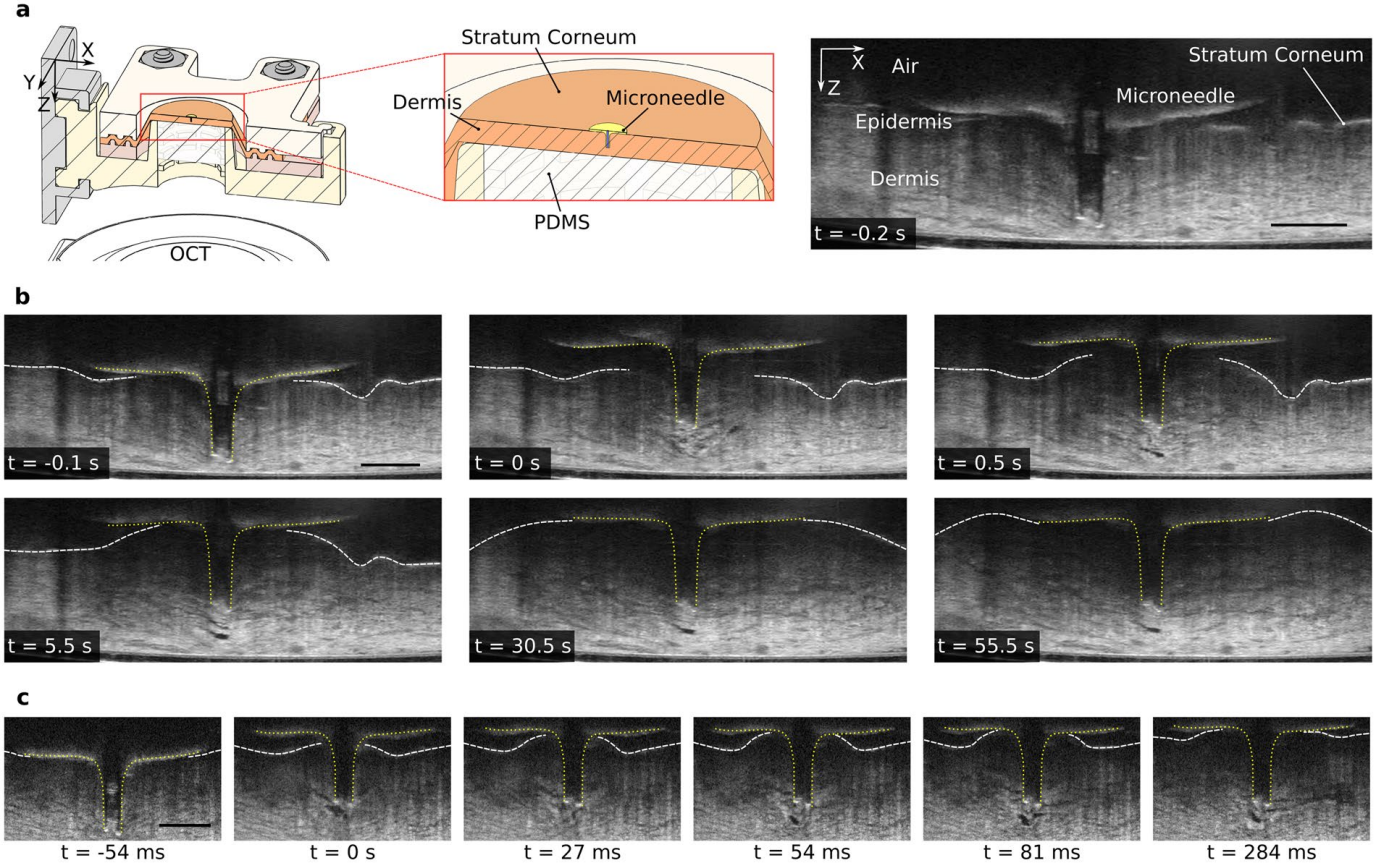
OCT images showing skin tissue deformation during the injection of water. (**a**) Cross-sectional schematic (mid-section) showing the microneedle inserted into the stretched skin sample (left) with OCT imaging the skin and the microneedle from the bottom through the transparent PDMS backing. OCT image (right) showing a cross-section of skin with the microneedle inserted, 0.2 s before retracting the microneedle, with fluid input pressure set to 100 kPa. (**b**) Sequence of OCT images (captured at 17.2 frames/s) showing the microneedle (yellow dotted lines) retracting by 0.3 mm at 0 s, and showing the deformation of skin tissue after 0 s. Skin tissue is stationary before retraction even after applying 100 kPa input pressure (as seen from the similar OCT images 0.2 s and 0.1 s before retraction). The surface of the skin, the stratum corneum (white dashed lines), moves upwards towards the base plate of the microneedle and then moves laterally outwards. Skin tissue absorbs water by locally expanding; water is transparent to OCT and appears as dark pixels. The region of injected water and tissue expansion grows, starting from a small region near the microneedle (real-time injection video in Supplementary Video 1). (**c**) OCT images captured at a higher frame rate (73.9 frames/s) than (**b**) showing skin tissue expansion during initial injection after retracting the microneedle by 0.3 mm at 0 s (Supplementary Video 2). The scale bars (black) in (**a**–**c**) are 0.5 mm.

The OCT measures the intensity of backscattered light; since water is transparent to the OCT, it only records the scattered light from skin tissue and not from the injected fluid. The greyscale intensity of skin tissue in an OCT image has sufficient contrast for tracking the expansion of tissue. Regions of only water appear dark due to the lack of scatter sources, allowing us to identify the formation of fluid-filled cavities. Before retracting the microneedle, the tissue structure in successive OCT images remained identical, even with the application of 100 kPa input pressure (Fig. 3a). The corresponding flow sensor measurements indicated that no fluid was injected into the tissue. The OCT images of microneedle insertion for most experiments showed that tissue deformed into the lumen of the microneedle, possibly plugging the flow and forming a mode II ring crack. Upon insertion, the base of the microneedle also represented a mechanical fixed boundary condition preventing the upward movement of the stratum corneum and the swelling of the skin tissue.

As opposed to pre-retraction, the tissue structure in corresponding OCT images post-retraction changed (Fig. 3b): the injected fluid deformed the tissue. The movement of the MN base removed the fixed boundary condition at the top and created an air gap, allowing the stratum corneum to move freely upwards and the tissue to swell. The skin tissue behaved like a deformable porous medium, as modelled by some researchers^17,18^. However, the fluid did not form a large cavity, which grew over the course of the injection, as suggested by Barry and colleagues^17^. Instead, the skin tissue expanded locally to absorb the injected fluid (Fig. 3b,c), for an input pressure of 100 kPa.

The OCT images showed the evolution of the tissue expansion and the location of the skin surface over time. Initially, the region of locally expanded tissue was limited to a small area near the MN tip, with the surrounding tissue structure remaining stationary. As the injection progressed, the initial region of expanded tissue stayed stationary while the adjacent tissue expanded; this region of expanding tissue moved away from the microneedle tip increasing the region of expanded tissue. The rate of tissue expansion differed between different parts of the injection (Supplementary Video 1). Initially, the tissue expanded rapidly, when the flow-rate was high (microinjection). Then as the free surface of the skin bulged upwards and reached the microneedle base, where it flattened and then grew laterally, the flow-rate decayed to the much lower steady-state value, which was associated with slow tissue expansion.

### 2D strain fields for quantifying tissue deformation

The OCT images were processed to extract other useful information such as the strain in tissue, the boundary between expanded and unexpanded tissue, and the total volume of tissue expansion. To quantify the tissue deformation and to relate it to the injected volume, we calculated the Green-Lagrangian strain in the tissue using digital image correlation (DIC) of the OCT images. We considered two settings: one with a higher frame rate (73.9 frames/s) and smaller region of interest (ROI) to obtain strain fields at high temporal resolution during initial injection, and another with a lower frame rate (17.2 frames/s) and larger ROI to compare results from flow sensor and OCT over a longer period (50 seconds). In both cases, the ROI excluded some parts of the upper dermis/epidermis where the contribution of noise was considerably higher than for the lower dermis because the OCT signal was attenuated by optical scattering. After obtaining the strain fields *E*_*xx*_, *E*_*xz*_, and *E*_*zz*_ in Cartesian coordinates from the DIC algorithm, we calculated the 2D strain fields:

1 $${\varepsilon }_{2D}={E}_{xx}+{E}_{zz}+{E}_{xx}{E}_{zz}-{E}_{xz}^{2}$$

**Figure 4:**
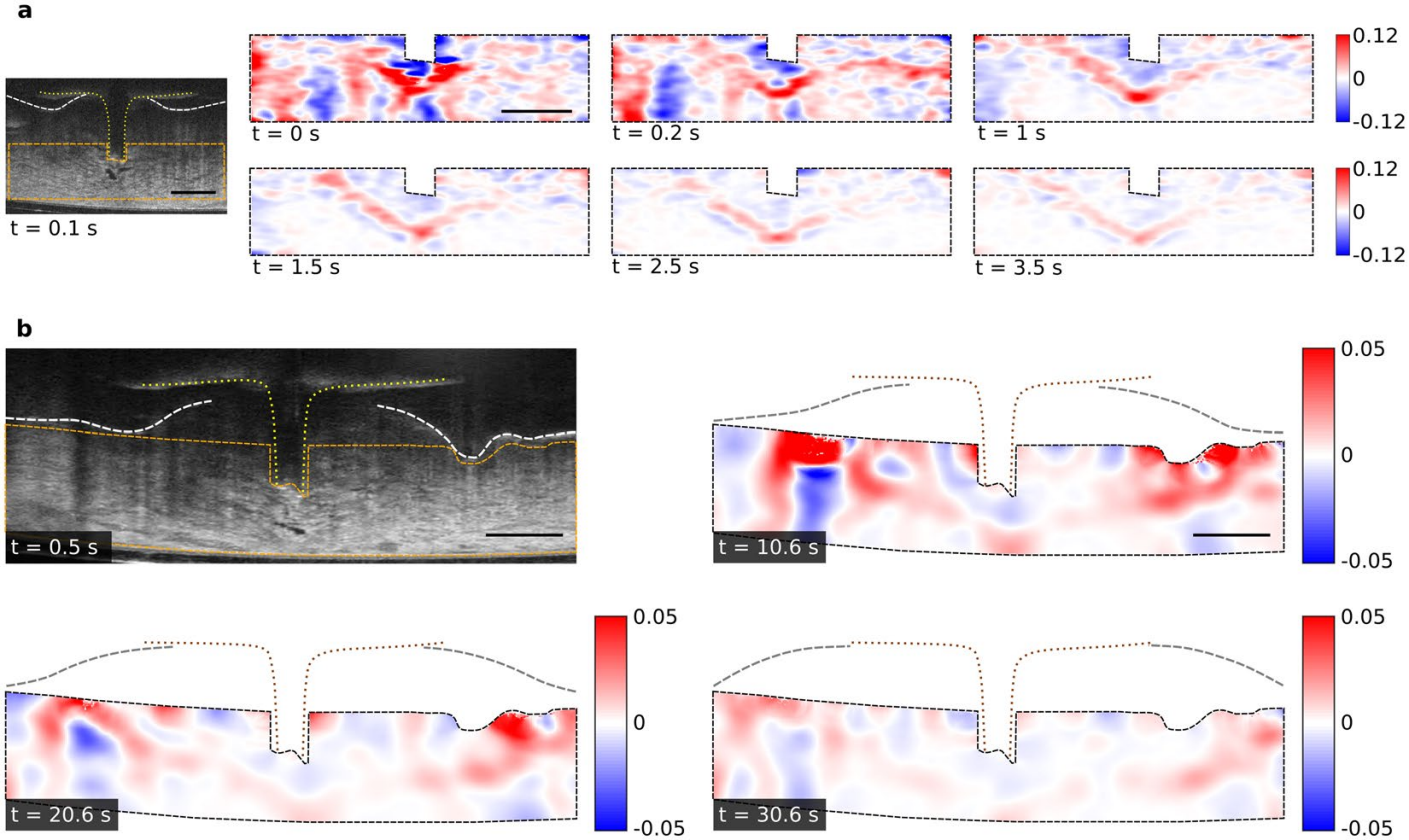
2D maps of strain showing local tissue deformation. (**a**) 2D maps of strain (*ε*_2*D*_) for the region bounded by orange dashed lines in the OCT image (left). Each strain map shows cumulative strain using DIC results on 8 pairs of consecutive (averaged) OCT images, with a total time difference between the reference OCT frame and the last OCT frame of 0.11 s (see Supplementary Fig. S5). Regions of local tissue expansion (positive strain) are shown in red, while regions of local tissue compression (negative strain) are shown in blue. A ‘V’ shaped region of high tissue expansion (red) grows over time, after retracting the microneedle at 0 s. OCT images captured at 73.9 frames/s; *P*_*in*_ = 100 kPa and *d*_*r*_ = 0.3 mm. (**b**) 2D maps of strain (*ε*_2*D*_) for the region bounded by orange dashed lines in the OCT image. The position of the microneedle is shown using yellow dotted lines in the OCT image and brown dotted lines in strain maps. The position of the skin surface is shown using white dashed lines in the OCT image and grey dashed lines in the strain maps. Each strain maps shows cumulative strain using DIC results on 9 pairs of consecutive OCT images, with a total time difference between the reference OCT frame and the last OCT frame of 0.52 s (see Supplementary Fig. S4). The ‘V’ shaped region of high tissue expansion grows laterally outwards. The rate of tissue expansion decreases over time as indicated by the decrease in strain magnitude over time (strain map for 0.6 s shown in Supplementary Fig. S8). OCT images captured at 17.2 frames/s; *P*_*in*_ = 100 kPa and *d*_*r*_ = 0.3 mm. The scale bars (black) in **a** and **b** are 0.5 mm.

The magnitude of 2D strain represents a relative increase/decrease in area of the corresponding interrogation window considered for the DIC algorithm, and provides a measure for local tissue expansion/compression in 2D. The strain magnitude is proportional to the rate of tissue expansion/compression because the time interval between OCT frames used for DIC is constant for each injection. The strain fields provide a distribution of tissue that is either locally expanding, compressing or stationary, with the magnitude representing the extent of tissue deformation – also proportional to the rate of deformation. The magnitude of strain was higher for the first few seconds than later during the injections (Fig. 4a), indicating a high initial rate of tissue deformation (during microinjection) followed by a lower rate (during microinfusion); positive values of strain represent regions of tissue expansion, while negative values represent regions of tissue compression.

Initially, the deformation of tissue was limited to a region near the MN tip. A ‘V’ shaped region of high positive 2D strain near the MN indicated the site where tissue absorbed fluid, by locally expanding (Fig. 4a). The ‘V’ shaped region of high positive strain, corresponding to high tissue expansion, increased in size with time, moving downwards and sideways (Fig. 4a). The initial strain fields during an injection indicated that initially tissue near the MN tip expanded locally, and then flow through this previously expanded tissue further expanded the surrounding tissue. Thus, the region of high tissue expansion, representing the boundary of absorbed fluid, moved away from the MN tip as an injection continued. We observed a similar decrease of strain magnitude and growth of the expansion region over a longer period (Fig. 4b). Here, the fixed lower boundary prevented the downward movement of the ‘V’ shaped boundary, such that the ‘V’ shape widened and moved mostly laterally outwards. In contrast to the results after retraction, the strain maps before retraction were similar to those before microneedle insertion, which indicated that the deformation of tissue due to applied pressure was negligible before retracting the microneedle (see Supplementary Fig. S7).

### Correlation maps for tracking the growth of tissue expansion

**Figure 5:**
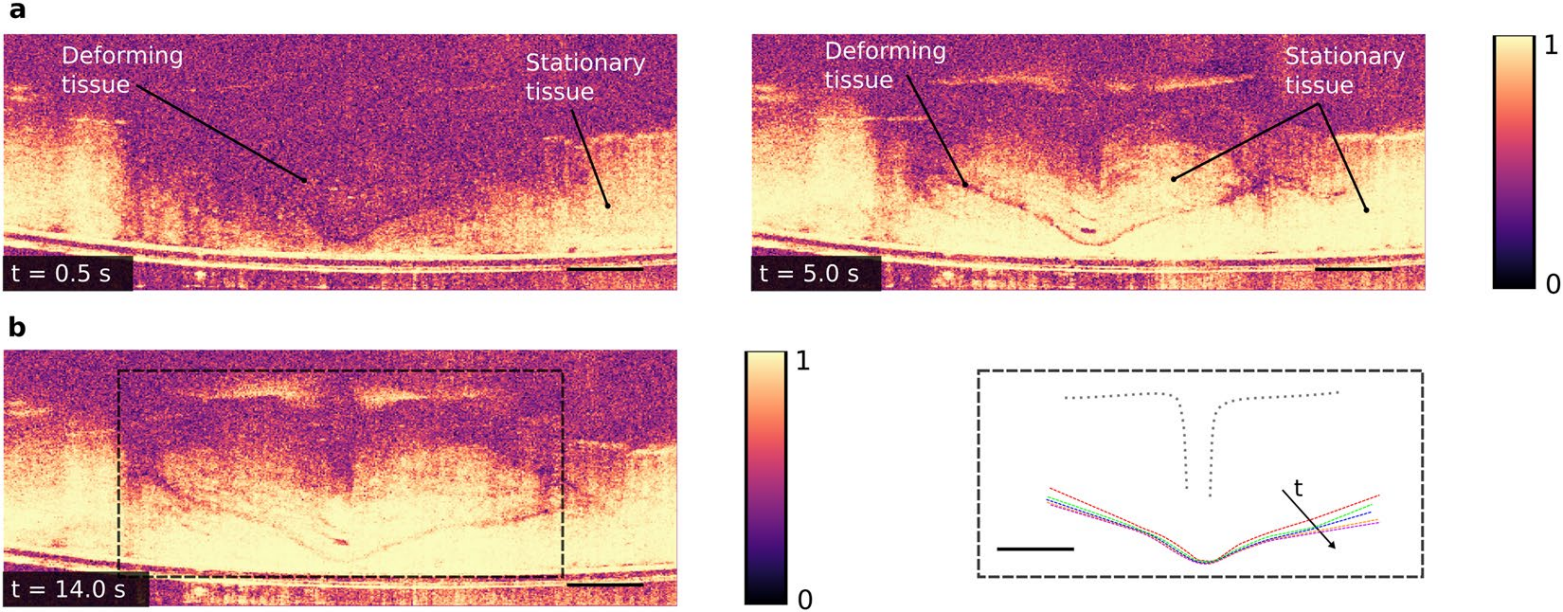
Correlation of subsequent images to qualitatively track the growth of tissue expansion. (**a**) High correlation coefficient (bright pixels) indicates stationary tissue, while low correlation coefficient (dark pixels) indicates deformation or noise. Left: 0.5 s after retraction, a major portion of the tissue is deforming (low correlation coefficient), while small regions of tissue far away from the microneedle tip are stationary. Right: 5.0 s after retraction, a thin region of tissue is deforming (separating the region of expanded tissue above and stationary tissue below). A major portion of the deforming tissue at 0.5 s turns stationary at 5.0 s; *P*_*in*_ = 100 kPa and *d*_*r*_ = 0.3 mm. (**b**) Left: Correlation map 14.0 s after retraction. Right: Evolution of the lower boundary between expanded and stationary tissue tracked over time, showing growth of the expanded region. Successive dashed (colored) lines are 2.9 s apart, with the first (red) dashed line starting 2 s after microneedle retraction; microneedle position shown by dotted gray line. The scale bars (black) are 0.5 mm.

To qualitatively observe the evolution of the boundaries between stationary tissue and deforming tissue, we computed correlation maps of the OCT images that provide 2D representations of how similar consecutive images are (Methods). In each correlation map, a high correlation coefficient, represented by bright pixels, corresponds to stationary tissue, while a low correlation coefficient, represented by dark pixels, corresponds to deforming tissue or noise (Fig. 5a). Unlike DIC, the correlation maps calculate only correlation coefficients (not strain) and are computationally faster to achieve. The correlation maps show the regions of motion more clearly than the strain maps, especially when the gradients of strain between expanding and stationary tissue are low, such as during microinfusion. The thin region of low correlation coefficient between regions of high correlation coefficient (Fig. 5a right) resembles the ‘V’ shaped region of high strain obtained from DIC, and the movement of this region over time qualitatively shows the growth of the expanded tissue region (Fig. 5b right). A large portion of the deforming tissue successively turns stationary (Fig. 5a and Supplementary Video 5) as the correlation coefficient changes from a low to a high value and the region of expanded tissue grows, indicating that fluid expands the tissue up to a maximum limit that is most likely given by the injection pressure. We observed similar results for the growth of the expanded region for multiple injection experiments (Supplementary Fig. S9 and Supplementary Video 6).

### Volume of Tissue Expansion

**Figure 6:**
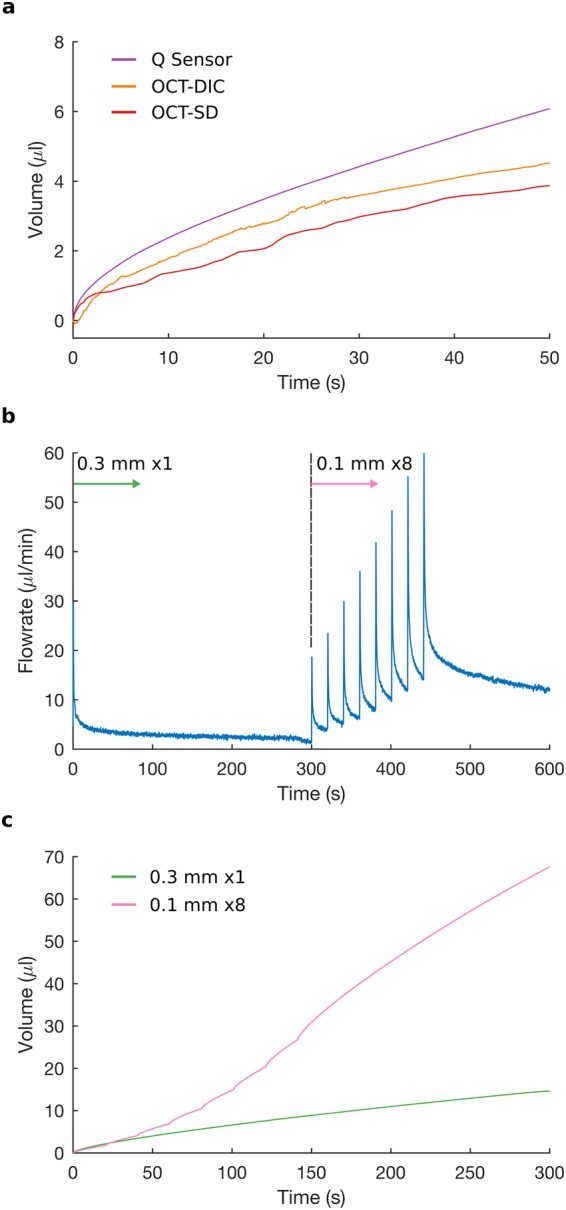
Volume of tissue expansion and the effect of successive retractions. (**a**) The time evolution of total volume of tissue expansion derived from 3D volumetric strain (orange curve; OCT-DIC) and from surface deformation technique (red curve; OCT-SD) have similar profiles to that of total injected volume (purple curve; Q Sensor). High fluid absorption, during microinjection, corresponds to high tissue expansion, and low fluid absorption, during microinfusion, corresponds to low tissue expansion. (**b**) Flow-rate after retraction of 0.3 mm at 0 s, followed by 8 retractions of 0.1 mm (spaced 20 s apart) starting at 300 s. After each retraction, flow-rate increases rapidly (microinjection) and then decays to a value greater than that of the previous retraction; *P*_*in*_ = 100 kPa. (**c**) Volume of fluid injected is higher for 8 successive retractions of 0.1 mm than for a single retraction of 0.3 mm (calculated by integrating the curve in (**b**) from 300 s to 600 s, and from 0 s to 300 s, respectively).

The total volume of tissue expansion was calculated from the OCT images using 3D volumetric strain and surface (SC) deformation (Supplementary Information), assuming tissue deformations recorded in 2D were actually axisymmetric around the central axis of the MN. The total volume of tissue expansion calculated using these two methods shows a similar trend as compared to that of the injected volume (Fig. 6a). A high rate of total tissue expansion and surface deformation indicated by a rapid increase in volume, corresponds to high rates of injected volume. Thus, high fluid absorption is associated with high local tissue expansion, as seen initially during microinjection. A similar link between fluid absorption and tissue expansion was previously observed by modelling skin tissue as deformable porous medium^17^, where fluid absorption by the tissue was associated with an overall expansion of the medium and an increase in the local permeability.

The volume of injected water calculated from the flow sensor was higher than the volume of tissue expansion calculated from strain or surface deformation. The volume of tissue expansion from strain was lower than the injected volume because the ROI for DIC was limited, not accounting for local tissue deformation outside the ROI. Both estimates, from strain and surface deformation, also ignored any flow through the un-deformed porous network, since the techniques only account for expanded tissue and cannot measure fluid flow directly.

### Multiple retractions increase fluid flow

We compared the injection following a single retraction of 0.3 mm to that associated with multiple successive retractions (8 retractions of 0.1 mm each, in 20 second steps). The behavior of fluid flow and tissue deformation after each successive retraction resembled that after a single retraction (Fig. 6b). After each successive retraction of 0.1 mm, the flow-rate increased rapidly and decayed towards a steady-state value higher than that after previous retractions. At each retraction, the fixed boundary condition at the stratum corneum was removed, which was followed by the swelling of the tissue due to fluid absorption and the upward movement of the stratum corneum to the MN base. Each time when the free surface reached the MN base, the MN base provided a mechanically fixed boundary, which limited tissue expansion in that direction. Thus, limiting/restricting tissue expansion reduces the fluid flow-rate or fluid absorption, and removing the fixed boundary restricting expansion – allowing tissue to expand – increases fluid flow successively.

The final steady-state flow-rate (and injection volume) following 8 successive retractions of 0.1 mm each was higher than that following a single retraction of 0.3 mm (Fig. 6b,c). Previous studies^11^ have shown that, for a single retraction, a larger retraction distance resulted in a higher injection flow-rate (and volume). Thus, we expected a cumulative retraction of 1.1 mm (0.3 mm for single retraction + 0.8 mm for multiple retractions) to result in a higher flow-rate than that due to a 0.3 mm retraction. However, a single (step) retraction of 1.1 mm was not possible because the height of the microneedle used in our experiments was 0.7 mm, and the microneedle would have retracted completely out of the skin. By retracting the microneedle successively, and allowing tissue to swell/expand after each retraction, we achieved a cumulative retraction higher than that possible by a single step retraction, resulting in a greater volume of injection. We achieved similar results after a (slow, i.e. 0.1 mm/s) continuous retraction of the microneedle to a total distance of 1 mm (Supplementary Fig. S10 and Supplementary Video 7), where continuous retraction of the microneedle resulted in a higher injection volume than a single step retraction.

## Conclusions

We identified two modes of flow during intradermal injections: microinjection at a high transient flow-rate, and microinfusion at a low steady state flow-rate. We demonstrated OCT as a suitable tool for visualizing tissue deformation during intradermal injections and a potential imaging modality for observing the dynamic behavior of biological tissue with micron-level resolution in real time. Skin tissue was found to absorb injected fluid by locally expanding, without forming a single large fluid-filled cavity. We quantified this local tissue deformation using Green-Lagrangian strains in 2D by performing DIC on OCT images, and calculated the total volume of tissue expansion using 3D volumetric strain. From the calculated volume of tissue expansion and the volume of injected fluid, we found that more fluid was injected when overall tissue expansion was high, indicating that skin tissue behaved like a deformable porous medium with variable permeability: local tissue expansion due to fluid absorption increased the porosity and permeability of tissue locally, thus increasing fluid flow through that region and aiding further fluid absorption in adjacent regions. We qualitatively tracked the growth of the region of tissue expansion and fluid absorption using correlation maps. We also observed that limiting the tissue expansion by the needle base, which formed a fixed boundary, reduced the fluid flow-rate into the tissue, and removing this fixed boundary increased the flow-rate. By taking a closer look at the dynamics of tissue deformation during intradermal injections and understanding the interactions between fluid and tissue, we can better design injection systems to deliver drugs at therapeutically relevant time scales. The injection protocol of drugs can be controlled based on the therapeutic requirement: a rapid bolus injection of drugs can be accomplished by allowing tissue to freely expand (e.g. by retracting MN successively or continuously, to prolong the microinjection mode); or a slow and controlled infusion of drugs can be accomplished by restricting tissue expansion (e.g. by retracting the MN in a single short step, to maintain the microinfusion mode). Our experimental results can also provide physical insights for mathematical modelling or numerical simulations of flow through biological tissue, especially for intradermal delivery of drugs into skin tissue.

## Methods

### Experimental Setup

We designed an experimental setup to observe the dynamics of intradermal injections. This setup allows impacting the microneedle onto the skin sample, retracting the microneedle, and controlling input fluid pressure. During each experiment, we visualized the deformation of skin tissue due to fluid flow, while continuously recording the flow conditions such as the pressure of the fluid as it enters the needle *P*_*m*_, and the flow-rate *Q*. To ensure repeatability between experiments, we controlled the following injection parameters: fluid input pressure *P*_*in*_, microneedle impact velocity *v*_*im*_, insertion depth *d*_*i*_, and retraction distance *d*_*r*_. A pressure controller supplied compressed air at *P*_*in*_ to a fluid reservoir containing the injection fluid, which flowed, through a flow-rate sensor and a pressure sensor, to a single hollow microneedle. An impact system and a retraction system controlled the vertical position of the microneedle during insertion and injection, respectively. A tissue holder secured a sample of excised porcine skin tissue, which was visualized, along with the microneedle, using optical coherence tomography.

We used a hollow microneedle, with a height of 700 μm and lumen diameter of 100 μm. The microneedle was fabricated by electroplating nickel onto polymeric (SU-8) pillars, as described by Mansoor *et al*.^33^. To deliver fluids into the dermis, the microneedle needs to first pierce the stratum corneum at a certain impact velocity *v*_*im*_, as reported by Verbaan *et al*.^43^ where a velocity of 1 or 3 m/s pierced human skin reproducibly. Our impact system uses the energy of a compressed spring with a spring constant k = 343 N/m to insert the microneedle at *v*_*im*_ of approximately 2 m/s into a sample of excised porcine skin tissue. The skin sample was stretched uniformly in radial directions in the tissue holder by a transparent polydimethylsiloxane (PDMS) backing. The PDMS (SYLGARD 182 Silicone Elastomer) was prepared by thoroughly mixing the base and curing agents at a 10:1 weight ratio, vacuum de-airing to remove bubbles, and pouring into a hollow cavity of an aged 3-D printed part made of Verowhite (Stratasys Direct, Inc.); the 3-D printed part was left at room temperature and pressure for 1 week, after which the material did not interfere with the curing process of the PDMS. The PDMS backing provided three main functions: stretching the skin sample back to its original dimensions; maintaining a fixed and impermeable boundary condition at the bottom surface of the dermis; and providing optical access for visualizing tissue deformation.

To capture tissue deformation during injections at sub-second temporal resolution and micron-level spatial resolution, we used the Thorlabs TELESTO-II Spectral Domain OCT Imaging System. The OCT uses near-infrared light, with a central wavelength of 1300 nm, to provide 2D cross-sectional images of the skin tissue. The PDMS is transparent to the OCT. The OCT has an axial resolution of 5.5 μm in air (4.2 μm in water), a lateral resolution of 13 μm and an imaging depth of 3.5 mm in air. In a highly scattering material such as skin tissue, the imaging depth is reduced to around 1–2.5 mm. The OCT images were recorded using a computer, and were processed and analyzed using MATLAB and ImageJ, as described later.

Apart from the OCT image acquisition, other injection parameters were also measured or controlled using a computer. A LABVIEW program (National Instruments, Austin, TX, USA) synchronized the control and measurement of the injection parameters and flow properties: controlling/measuring the input pressure *P*_*in*_; measuring the fluid flow-rate *Q*; measuring the fluid pressure *P*_*m*_; and controlling/measuring the retraction distance *d*_*r*_ (Fig. 1a). An input pressure *P*_*in*_ between 0–200 kPa was applied to the fluid reservoir by the pressure controller, Elveflow OB1. We used Milli-Q water, EMD Millipore Corporation, as the injection fluid, which was degassed for 3 minutes before each experiment. The flow-rate *Q* and pressure *P*_*m*_ of the injection fluid were measured at 100 Hz using an Elveflow Microfluidic Flow Sensor and an Elveflow Pressure Sensor, respectively. The retraction distance *d*_*r*_ was set using a linear motorized stage, Zaber T-LSM050A, with a microstep size (default resolution) of 0.048 μm. Some of the injection parameters, such as the microneedle impact velocity *v*_*im*_ and the insertion depth *d*_*i*_, were manually controlled. The impact velocity *v*_*im*_ was set using the compression of the spring, and our impact system gave a choice of three impact velocities *v*_*im*_ of 1.3 m/s, 2.0 m/s, and 2.6 m/s based on the three levels of spring compression 10 mm, 15 mm, and 20 mm. The insertion depth of the microneedle was controlled by vertically moving the tissue holder, which was mounted on a single-axis translation stage (z-axis) with a standard micrometer.

### Skin Sample Preparation

Porcine skin is often used as a model for human skin due to similarities in anatomy and physiology, and porcine skin has been found to have similar dermal/transdermal absorption as that of human skin^44^. For our experiments, excised porcine skin tissue from the abdomen of a female Yorkshire pig was received from the Center of Comparative Medicine (CCM) at the University of British Columbia. The use of animals was approved by the University of British Columbia’s Animal Care Committee. All experimental protocols conformed to the Canadian Council on Animal Care (CCAC) guidelines, and the methods were carried out in accordance with the CCAC guidelines. Freshly excised abdominal skin samples were cut into 30 *mm* × 25 *mm* rectangular pieces and frozen at −80 °C. Before each experiment, the frozen skin samples were thawed and sliced to a thickness of around 1–2 mm by removing subcutaneous fat using surgical scalpels and scissors. Removing the subcutaneous layer was necessary for visualizing the dermis and epidermis using OCT, because of its imaging depth of approximately 1–2.5 mm in biological media. Finally, to account for the contraction of the skin samples post excision to approximately 50% of their *in-vivo* area^45^, the skin was stretched uniformly in radial direction in the tissue holder to its approximate original size. Moisture treatment of the skin samples was not performed before an experiment, and the indoor relative humidity, which was not controlled, ranged between 25% to 40% for all the experiments.

### Injection Procedure

After preparing the porcine skin sample, it was mounted onto the tissue holder. The position of the tissue holder was adjusted such that the microneedle base aligned with the initial position of the stratum corneum, after microneedle impact when the spring was relaxed. This adjustment led to consistent insertion depths between experiments, and prevented the microneedle base from applying additional compressive stresses to the skin at impact. At the pre-impact position, with the spring compressed and the microneedle raised, pressure was applied to the fluid to fill the sensors, microneedle and tubing. After expelling water out of the microneedle for a few seconds, the microneedle impacted the skin sample to penetrate the stratum corneum. A constant input pressure, *P*_*in*_ = 100 *kpa*, was then applied to the fluid using the OB1 pressure controller. The application of input pressure was followed by a rapid increase in flow-rate due to the compliance of the tubing in the system followed by a rapid decay to zero flow-rate. A delay of 10 seconds before beginning the retraction protocol ensured that the tubing compliance did not affect flow-rate readings during the experiment. The OCT captured cross-sectional images at the approximate mid-section of the microneedle. The movement of the skin tissue and the microneedle were observed during fluid injection.

### OCT Image Acquisition

The OCT creates a 1D depth profile of the sample using the interference pattern from the backscattered light (from the sample) and a reference light beam. The system combines multiple 1D scans to form a 2D cross-sectional image and successive 2D tomographic sections can be stacked to provide a 3D image of the sample^25^. To capture the injections at a high frame rate, only one 2D cross-section of the sample (passing through the approximate center of the microneedle) was acquired over time. In each cross-sectional OCT image, the OCT signal attenuates as the near-infrared light moves deeper into the tissue (towards the microneedle base) through multiple scattering sources in the tissue. The acquisition time of an OCT frame, which corresponded to a single cross-section of the skin, depended on the selected axial scan rate, axial scan averaging, field of view (FOV) and pixel resolution. In our experiments, the acquisition time for each OCT frame was either 58 ms (Inj1) or 13.5 ms (Inj2) depending on the scan parameters as shown in Supplementary Table S2, corresponding to frame rates of either 17.2 frames/s or 73.9 frames/s, respectively. The OCT records the structure of the skin tissue and the movement of tissue, while not recording the movement of fluid. The movement of a transparent fluid, like water, can only be recorded by seeding the fluid with particles and recording the movement of particles. However, the particles represent scattering sources that create speckles in an OCT image, just as scattering sources in the skin tissue create speckles. In our preliminary investigation of injecting fluid seeded with particles (500 nm; 0.2% concentration by weight), we could not clearly identify whether regions of speckle movement were a result of moving particles (in the fluid) or deforming tissue. Therefore, in our final experiments we injected a transparent fluid, water, to only track the movement of the tissue.

Due to the difference in the refractive indices of air and skin, the vertical dimensions of the skin and its features in an OCT image differ from their actual dimensions. Near infrared light, used by OCT, travels faster in air than in skin because the refractive index of air is lower than that of skin. For a given time, the distance travelled by light in the reference medium (air) is larger than the distance travelled in the sample (skin). Therefore, the samples appear to be elongated vertically in the OCT images by a factor equal to the refractive index of the material (skin), assuming the refractive index of air is unity. The OCT images were adjusted by dividing the vertical dimension of the image by the refractive index of the skin (*n*_*skin*_), estimated to be 1.375 ± 0.006 (see Supplementary Fig. S2). Since the entire image was scaled based on the refractive index of skin, the distances in the skin in the OCT images represented actual physical distances in skin and the distances in air in the same OCT images were reduced by a factor equal to *n*_*skin*_. This reduction of distance in air did not affect any results, since all the analysis included distances in the skin.

### Speckle Reduction in OCT Images

OCT uses interferometry as its measurement technique and relies on the coherence (spatial and temporal) of optical waves backscattered from the sample. However, in a highly scattering material such as skin tissue, multiple back scattering and forward scattering events of the beam combine randomly to create speckle noise in the interference signal^46^. Speckle noise reduces the signal-to-noise ratio and the contrast of OCT images^47^, degrades image quality by producing grainy appearances^48^, and limits the performance of subsequent quantitative image analysis^49^. We used two different methods for reducing speckle noise in our OCT images: averaging consecutive OCT images, and wavelet-transform based filtering (see Supplementary Fig. S3).

Some researchers^50^ have averaged axial scans of OCT for real-time reduction of speckle noise, but here we averaged OCT images post-acquisition. The same speckle noise reduction could have been achieved by longer averaging during OCT recording, but that would have reduced the temporal resolution. Here, we maintain the temporal resolution of 13.5 ms while reducing speckle noise: we average images n-1 and n to image A and average images n and n + 1 to image B while maintaining the inter-image period. Averaging was only used for OCT images captured at high frame rates (Inj2–73.9 frames/s), since at lower frame rates (Inj1–17.2 frames/s) averaging produced significant motion artifacts. The greyscale intensity at each pixel location in an image was averaged with that of the corresponding pixel location in the consecutive image. The averaged images were used to quantify tissue deformation using Digital Image Correlation (DIC). For the high frame rate images, tissue deformations were captured at sufficiently high temporal resolution such that features of the tissue were not blurred by averaging.

For OCT images captured at 17.2 frames/s (Inj1), we used wavelet-transform based filtering to reduce speckle noise. Wavelet filtering has been found to significantly reduce speckle noise and increase the signal-to-noise ratio in OCT images, while preserving strong edges and maintaining image sharpness^48^. Transform domain filtering, such as wavelet and curvelet^49^ filtering, relies on the sparse representation of signal and widespread distribution of noise in the decomposition levels of the transform domain, and appropriate thresholds that attenuate noise. The wavelet denoising procedure, similar to earlier implementations^47,48,51^, included five main steps: logarithmic conversion, forward wavelet transform, thresholding, inverse wavelet transform, and exponential conversion. Logarithmic transformation (base 10) of the OCT image converted speckle noise, modelled usually as multiplicative noise, into additive noise. Then, a multi-level wavelet decomposition of the logarithmic image produced wavelet coefficients at different decomposition levels (up to level N). The signal was first decomposed into a low pass sub-band (approximation level) and a high-pass sub-band (detail-level), and the approximation level was further decomposed for multi-level analysis. The 2D maps of the detail coefficients at horizontal, diagonal and vertical subbands were visually analysed to adjust thresholds at different decomposition levels. A higher threshold was applied to 2D maps that had detail coefficients predominantly represented in regions of high noise outside the skin, where most of the OCT signal was attenuated. Inverse wavelet transform reconstructed the denoised image, and an exponential conversion (base 10) changed the image from the logarithmic scale to the original linear scale. We used the 2D Stationary Wavelet Transform function from the Wavelet Toolbox in MATLAB for denoising images. We applied a near symmetric, eight-tap symmlet wavelet (*sym-8*) with a four-level decomposition (N = 4) and soft thresholding with a greater threshold applied to vertical subbands to preserve horizontal edge information common in OCT images of tissue, as described by Adler and colleagues^48^.

### Digital Image Correlation

Digital Image Correlation (DIC) is a non-contact technique for measuring material deformation, with a wide variety of applications including multiscale biomechanics^52^. For measuring tissue deformation in our OCT images, we used NCorr, an open-source 2D subset-based DIC software package implemented in MATLAB^53^. In subset-based algorithms, a reference image is partitioned into subsets or subwindows, smaller regions that are initially contiguous groups of points. Deformations inside each subset are assumed to be homogenous, and deformed subsets are tracked in a current image. NCorr calculates Green-Lagrangian strains, based on four displacement gradients:

2 $${E}_{xx}=\frac{1}{2}(2\frac{\partial {u}_{x}}{\partial x}+{(\frac{\partial {u}_{x}}{\partial x})}^{2}+{(\frac{\partial {u}_{z}}{\partial x})}^{2})$$

3 $${E}_{xz}={E}_{zx}=\frac{1}{2}(\frac{\partial {u}_{x}}{\partial z}+\frac{\partial {u}_{z}}{\partial x}+\frac{\partial {u}_{x}}{\partial x}\frac{\partial {u}_{x}}{\partial z}+\frac{\partial {u}_{z}}{\partial x}\frac{\partial {u}_{z}}{\partial z})$$

4 $${E}_{zz}=\frac{1}{2}(2\frac{\partial {u}_{z}}{\partial z}+{(\frac{\partial {u}_{x}}{\partial z})}^{2}+{(\frac{\partial {u}_{z}}{\partial z})}^{2})$$

NCorr uses its strain window algorithm to compute the displacement gradients and Green-Lagrangian strains, and obtains the entire strain field of the selected ROI (see Supplementary Fig. S6). We used NCorr to calculate strain fields for two cases: averaged (Inj2), and wavelet filtered (Inj1).

### Volume of Tissue Expansion

To see how tissue expansion relates to fluid absorption, we estimated the total volume of tissue expansion using OCT images and compared it with the total injected volume from sensor measurements. We calculated the volume of tissue expansion from OCT images using two methods: surface deformation and volumetric strain. The surface deformation technique considered the swelling of the entire porous medium – a macroscopic view of expansion of the whole skin tissue. Assuming incompressibility of individual (solid and fluid) components of tissue, any fluid added by injecting through the MN inflates the porous medium. Thus, the volume of the injected fluid at any instant is equal to the increased bulk volume of the porous medium with respect to its initial un-deformed bulk volume. We tracked this change in bulk volume of the medium by tracking the change in position of the top skin surface, with respect to its un-deformed position. We used ImageJ to outline the top surface of the skin and MATLAB to calculate the volume (Supplementary Information).

On the contrary, the volumetric strain technique considered local tissue expansion quantified using strain fields – a microscopic view of the expansion. The Green-Lagrangian strain fields calculated using DIC in 2D Cartesian coordinates were translated into 3D volumetric strain fields in cylindrical coordinates, which were used to calculate the total volume of tissue expansion (derivation in Supplementary Information). We determined the volume of tissue expansion, using both the techniques, for each OCT image of Inj1 that provided a volume estimate for the two techniques every 58 ms. Since each OCT image was a 2D cross-sectional image at the approximate mid-section of the microneedle, we estimated the volume by assuming the tissue structure and its deformations were axisymmetric around the central axis of the microneedle. This axisymmetric assumption allowed us to estimate the total volume of tissue expansion at a rate equal to the frame rate of OCT acquisition.

### Correlation Maps

Each OCT image was divided into square interrogation windows of edge length 3 pixels, and 2D correlation coefficients between interrogation windows in consecutive images were calculated (using the corr2 function in MATLAB). To reduce the effect of noise, a 3-point running average was taken of 3 consecutive correlation maps. A correlation coefficient of 1 indicated that the image segments were identical (or tissue was stationary), while a value of 0 or -1 indicated that the image segments were completely uncorrelated or completely anti-correlated, respectively. The distribution of correlation coefficients was scaled between 0 and 1 to a 16-bit grayscale image, with bright pixels representing stationary tissue and dark pixels representing deforming tissue or noise. While noise was more prominent in the upper dermis, where the OCT signal was attenuated due to optical scattering, the correlation maps in the lower dermis distinguished stationary tissue from deforming tissue. We marked the approximate region of deforming tissue between regions of stationary tissue to qualitatively track the evolution of this boundary during the injection.

## Electronic supplementary material

Supplementary Information

Supplementary Video 1: OCT video (Inj1 – Real-time)

Supplementary Video 2: OCT video (Inj2 – Real-time)

Supplementary Video 3: OCT video (Inj1–10x slower)

Supplementary Video 4: OCT video (Inj2–10x slower)

Supplementary Video 5: Correlation maps video (Inj1 – Real-time)

Supplementary Video 6: Correlation maps video (Inj2 – Real-time)

Supplementary Video 7: Slow continuous retraction of 1 mm

## Data Availability

The data generated or analysed during this study are included in this published article (and its Supplementary Information files). Custom MATLAB codes and raw OCT files are available from the corresponding author on reasonable request.
