# Supplementary material for: Fluid absorption by skin tissue during intradermal injections through hollow microneedles
Source: Sci Rep. 2018 Sep 13;8:13749. doi: 10.1038/s41598-018-32026-9 (PMC6137045; doi:10.1038/s41598-018-32026-9)
Supplement: Supplementary file 1 — Supplementary Information [file 41598_2018_32026_MOESM1_ESM.pdf]

Fluid absorption by skin tissue during intradermal injections through hollow microneedles

Pranav Shrestha[1], Boris Stoeber[1,2]*

[1]Department of Mechanical Engineering, The University of British Columbia, 2054-6250 Applied Science Lane, Vancouver, British Columbia V6T 1Z4, Canada
[2]Department of Electrical and Computer Engineering, The University of British Columbia, 2332 Main Mall, Vancouver, British Columbia V6T 1Z4, Canada

*Corresponding author; email: boris.stoeber@ubc.ca

Contents

1 Supplementary videos 2
1.1 Supplementary Video 1: OCT video (Inj1 – Real-time) 2
1.2 Supplementary Video 2: OCT video (Inj2 – Real-time) 2
1.3 Supplementary Video 3: OCT video (Inj1 – 10x slower) 2
1.4 Supplementary Video 4: OCT video (Inj2 – 10x slower) 2
1.5 Supplementary Video 5: Correlation maps video (Inj1 – Real-time) 2
1.6 Supplementary Video 6: Correlation maps video (Inj2 – Real-time) 2
1.7 Supplementary Video 7: Slow continuous retraction of 1 mm 2
2 Volume of tissue expansion 3
2.1 Surface deformation technique 3
2.2 3D Volumetric strain 4
3 Estimating refractive index of skin using OCT 5
4 Sample OCT image processing 6
5 Time difference between DIC results 7
5.1 Inj1 7
5.2 Inj2 7
6 Sample DIC data 9
7 Strain fields before/after insertion and retraction 10
8 Strain map for initial injection 12
9 Correlation maps 13
10 Continuous retraction 14
11 Supplementary tables 16

1 Supplementary videos

1.1 Supplementary Video 1: OCT video (Inj1 – Real-time)

Video of injection of water into excised porcine skin tissue in real-time (17.2 frames/s), with scan parameters listed in Table S2. The scale bar in the video is 0.5 mm. The skin tissue expands rapidly after retracting the microneedle and the stratum corneum moves to the base of the microneedle. The tissue expansion slows down during microinfusion.

1.2 Supplementary Video 2: OCT video (Inj2 – Real-time)

Averaged OCT images of injection into excised porcine skin tissue in real-time (73.9 frames/s). The scale bar in the video is 0.5 mm. At high frame rates, the initial tissue expansion right after retraction can be captured (slowed version in Supplementary Video 1.4).

1.3 Supplementary Video 3: OCT video (Inj1 – 10x slower)

Video of initial injection of water into excised porcine skin tissue slowed down 10 times (played at 1.72 frames/s). The scale bar in the video is 0.5 mm.

1.4 Supplementary Video 4: OCT video (Inj2 – 10x slower)

Initial injection into excised porcine skin tissue slowed down 10 times (played at 7.39 frames/s). The scale bar in the video is 0.5 mm.

1.5 Supplementary Video 5: Correlation maps video (Inj1 – Real-time)

Correlation maps showing stationary tissue (high correlation) with bright pixels and deforming tissue or noise (low correlation) with dark pixels. The scale bar in the video is 0.5 mm. After retraction, the majority of tissue is deforming, which subsequently turns stationary. A thin band of deforming tissue moves away from the microneedle, spreading the region of fluid injection. Fig. 5 in the main text contains results from this data.

1.6 Supplementary Video 6: Correlation maps video (Inj2 – Real-time)

Correlation maps for Inj2 showing similar time evolution of deforming tissue as Video 1.5. The 'V' shaped region of deforming tissue results from the mechanically fixed boundary below the dermis and the compression of tissue. The scale bar in the video is 0.5 mm.

1.7 Supplementary Video 7: Slow continuous retraction of 1 mm

This video shows a demonstration of a slower continuous retraction of 1 mm, as compared to the single step retractions of 0.3 mm mostly discussed in the paper. The injected fluid, water, is mixed with green dye for demonstrating the uptake of fluid from the outside. Green dye was not used for the other experiments described in the paper. The injection pressure was set to 100 kPa. This video demonstrates a continuous retraction of 1 mm, which would not have been possible with a single step retraction, since the height of the microneedle used was 0.7 mm. Like the successive retractions described in the paper, the continuous retraction allows the tissue to swell as the microneedle is retracted, prolonging the microinjection mode of flow and resulting in a greater flowrate (and volume) than a single retraction case. The continuous retraction case is discussed further in section 10 of the Supplementary information.

2 Volume of tissue expansion

The following assumptions are used:

1. Skin is a deformable porous medium containing fluid filled pores (same as the injected fluid, water). Thus, the porous medium has only two components – fluid and solid
2. Individual components (solid and fluid) are incompressible
3. During injection, fluid is added at the source position (tip of the microneedle), with the total volume of solid remaining the same (no solid components added)

Consider two cases:

- A: Before injection (original un-deformed state of the porous medium)
- B: During injection and addition of fluid components

The volume of the porous medium is the sum of volumes of its fluid and solid components:

$$V_A = V_{A,f} + V_{A,s}\ ;\ \ V_B = V_{B,f} + V_{B,s} \tag{S1}$$

Taking the difference of the volumes and using assumption 3, we get:

$$V_B - V_A = V_{B,f} - V_{A,f} \tag{S2}$$

For $V_{B,f} > V_{A,f}$ (as in the case of injection of water in our experiments), the left-hand term is the overall expansion/swelling of the porous medium (change in total volume), which is equal to the injected volume of fluid (right hand term). From the OCT images, we calculate the overall expansion ($V_B - V_A$) using the surface deformation (SD) technique, and we calculate the local expansion using 3D volumetric strain.

2.1 Surface deformation technique

The overall expansion of the medium is calculated using the un-deformed (before injection) and deformed positions of the stratum corneum in the OCT images (Fig. S1). The change in area (using the 2D OCT images) is assumed to be axisymmetric around the central axis of the microneedle.

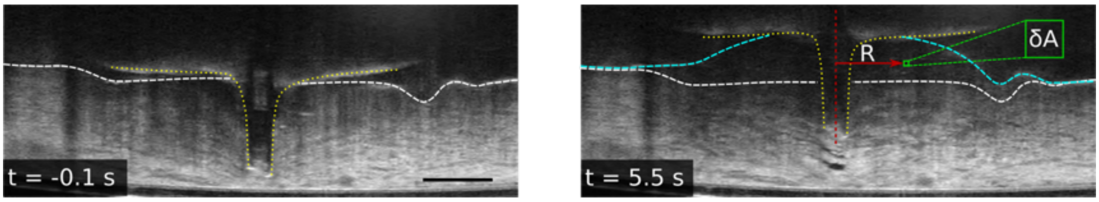

Fig. S1 | Surface deformation technique. Left: OCT image before injection, showing the un-deformed state of the skin that is used as a reference. The surface of the skin, the stratum corneum, is shown in white dashed lines and the microneedle is shown in yellow dotted lines. Right: OCT image during injection, showing the new position of the stratum corneum (blue dashed line), and the original un-deformed position (white dashed line). The area between the blue and white dashed lines is the added area, which is used for calculating the volume assuming axisymmetric conditions around the central axis of the microneedle (red dashed line). The scale bar (black) is 0.5 mm.

The volume of tissue expansion using the surface deformation technique is calculated as follows:

$$V_{SD} = \sum_i \sum_j \pi R_{ij} \delta A_{ij} \quad \text{(S3)}$$

Here, i and j span the entire domain between the blue and white curves (in Fig. S1) and would have a different value for a different pixel location in the OCT image. The radius (in mm) is denoted by R_{ij}, the area (in mm^2) is denoted by δA_{ij} (corresponding to an area equivalent to 1 pixel × 1 pixel), and V_{SD} is the volume of tissue expansion (in mm^3 or µl), which is equivalent to $V_B - V_A$ in equation (S2).

2.2 3D Volumetric strain

The strain fields obtained using digital image correlation on OCT images is used to calculate volume of tissue expansion. The volumetric strain, ε_V, is expressed as a ratio of the change in volume to the original volume:

$$\varepsilon_V = \frac{\Delta V}{V} \quad \text{(S4)}$$

The 2D strains and deformations in Cartesian coordinates are used to calculate the 3D volumetric strain in cylindrical coordinates. The 3D volumetric strain is then used to calculate the volume of tissue expansion. The x- and z- directions of the OCT cross-section in Cartesian coordinates are taken to be aligned with the r- and z- directions in the cylindrical coordinates (with z- axis aligned with the central axis of the microneedle). The change in volume (or expansion of tissue) is calculated using volumetric strain as follows:

$$\Delta V = \pi \sum_i \sum_j \varepsilon_{V,ij} r_{ij} \delta r \delta z \quad \text{(S5)}$$

Here, i and j span the region of interest chosen for the DIC algorithm, and would have different values for different subsets used for DIC. The change in volume, ΔV, is calculated for each subset and the values of δr and δz depend on the subset spacing chosen for DIC. The distance between the central axis of the microneedle and the subset origin is denoted by r_{ij}. The 3D volumetric strain for each subset is calculated using the sum of strain invariants (I_1, I_2, I_3):

$$\varepsilon_V = I_1 + I_2 + I_3 \quad \text{(S6)}$$

$$I_1 = \varepsilon_{rr} + \varepsilon_{zz} + \varepsilon_{\theta\theta} \quad \text{(S7)}$$

$$I_2 = \varepsilon_{rr}\varepsilon_{zz} + \varepsilon_{zz}\varepsilon_{\theta\theta} + \varepsilon_{\theta\theta}\varepsilon_{rr} - {\varepsilon_{rz}}^2 - {\varepsilon_{z\theta}}^2 - {\varepsilon_{\theta r}}^2 \quad \text{(S8)}$$

$$I_3 = \varepsilon_{rr}\varepsilon_{zz}\varepsilon_{\theta\theta} - \varepsilon_{rr}{\varepsilon_{z\theta}}^2 - \varepsilon_{zz}{\varepsilon_{\theta r}}^2 - \varepsilon_{\theta\theta}{\varepsilon_{rz}}^2 + 2\varepsilon_{rz}\varepsilon_{z\theta}\varepsilon_{\theta r} \quad \text{(S9)}$$

Using the axisymmetric assumption, where $u_\theta = 0$ and $\frac{\partial}{\partial\theta}[\,] = 0$, the 3D volumetric strain in cylindrical coordinates is given by:

$$\varepsilon_V = \varepsilon_{rr} + \varepsilon_{zz} + \varepsilon_{\theta\theta} + \varepsilon_{rr}\varepsilon_{zz} + \varepsilon_{zz}\varepsilon_{\theta\theta} + \varepsilon_{\theta\theta}\varepsilon_{rr} - {\varepsilon_{rz}}^2 + \varepsilon_{rr}\varepsilon_{zz}\varepsilon_{\theta\theta} - \varepsilon_{\theta\theta}{\varepsilon_{rz}}^2 \quad \text{(S10)}$$

The components ε_{rr}, ε_{zz} and ε_{rz} in cylindrical coordinates correspond to E_{xx}, E_{zz} and E_{xz} in Cartesian coordinates derived from DIC. The value of $\varepsilon_{\theta\theta}$ is given by $\frac{u_r}{r}$ for an axisymmetric case, and u_r can be related to the displacement u_x derived from DIC.

3 Estimating refractive index of skin using OCT

Due to the difference in refractive indices of skin and air, the height of skin in an OCT image is larger than its actual height (Fig. S2B). The refractive index of the skin can be estimated by considering an edge of a skin sample between two parallel glass slides (Fig. S2A). The air gap seen in the OCT image is not distorted because the reference medium is air.

The refractive index of the skin (n_{skin}) can be calculated using the heights of skin and air in the OCT image:

$$n_{skin} = \frac{h_{Skin}}{h_{Air}} \tag{S11}$$

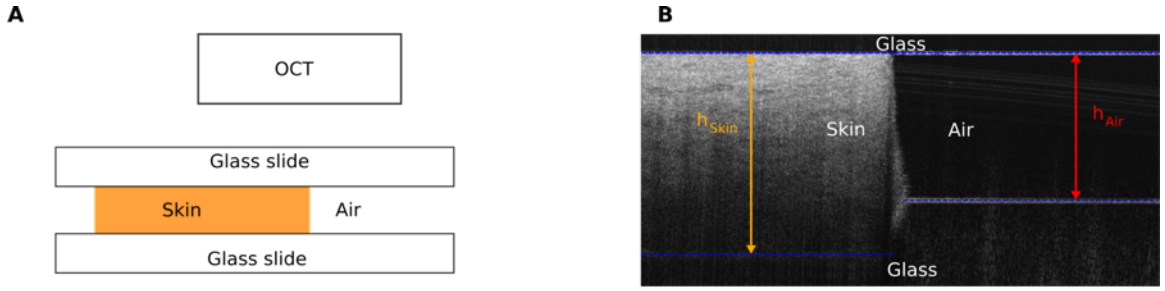

Fig. S2 | Refractive index of skin. A, Setup for measuring the refractive index of skin. **B**, The height of the skin in the OCT image appears larger because the refractive index of skin is higher than that of air.

We determined the refractive index of skin to be 1.375 ± 0.006 (s.d.), considering 23 OCT images. This estimate of refractive index is used to adjust the sizes of OCT images displayed in the paper, and to correct the vertical distances used for calculations.

4 Sample OCT image processing

In Fig. S3, a sample OCT image (I) is first adjusted to account for the skin's refractive index (IIa), and is denoised using either wavelet transform based filtering (IIIa) or averaging (IV). For displaying the images more clearly, the brightness can be adjusted in ImageJ as shown in IIb and IIIb. The OCT images presented in the paper are in the form IIIb (Inj1) or IV (Inj2).

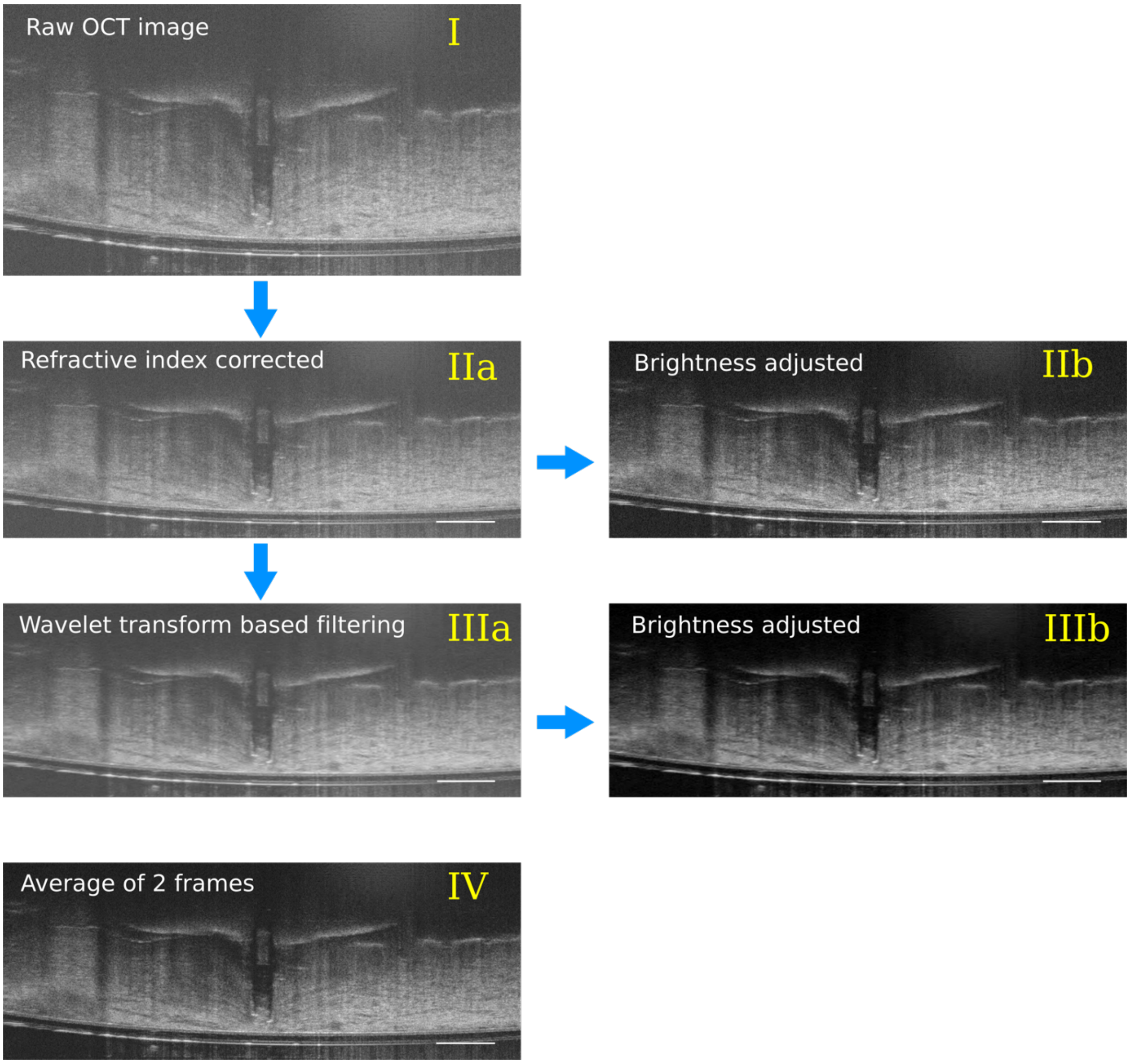

Fig. S3 | OCT image processing. Image processing of a sample OCT image, showing the different types of techniques. In the paper, OCT images for Inj1 and Inj2 are presented in the forms IIIb and IV, respectively. The scale bars are 0.5 mm.

5 Time difference between DIC results

The displacement fields and strain fields presented in the paper are not simply based on the DIC results on a pair of OCT images. The results displayed are cumulative results from 9 pairs and 8 pairs of images for Inj1 and Inj2, respectively.

5.1 Inj1

For Inj1, the procedure for performing DIC, as shown in Fig. S4, was as follows:

- Wavelet transform based filtering: Each raw OCT image (R1 – R10) was denoised using the technique described in Methods (image processing IIIa in Fig. S3). The time difference between consecutive denoised images (WD1 – WD10) was the same as that between the raw images (Δt = 58 ms).
- DIC on consecutive images: DIC was performed on consecutive denoised images (WD1 and WD2, WD2 and WD3, and so on), with the reference image being updated after each iteration. The time difference (Δt) between consecutive results was 58 ms.
- Cumulative DIC results: The DIC results from 9 pairs of images (a total of 10 images) were combined in the NCorr software to provide u_x, u_z, E_{xx}, E_{xz} and E_{zz} with a total time difference of 0.52 s.

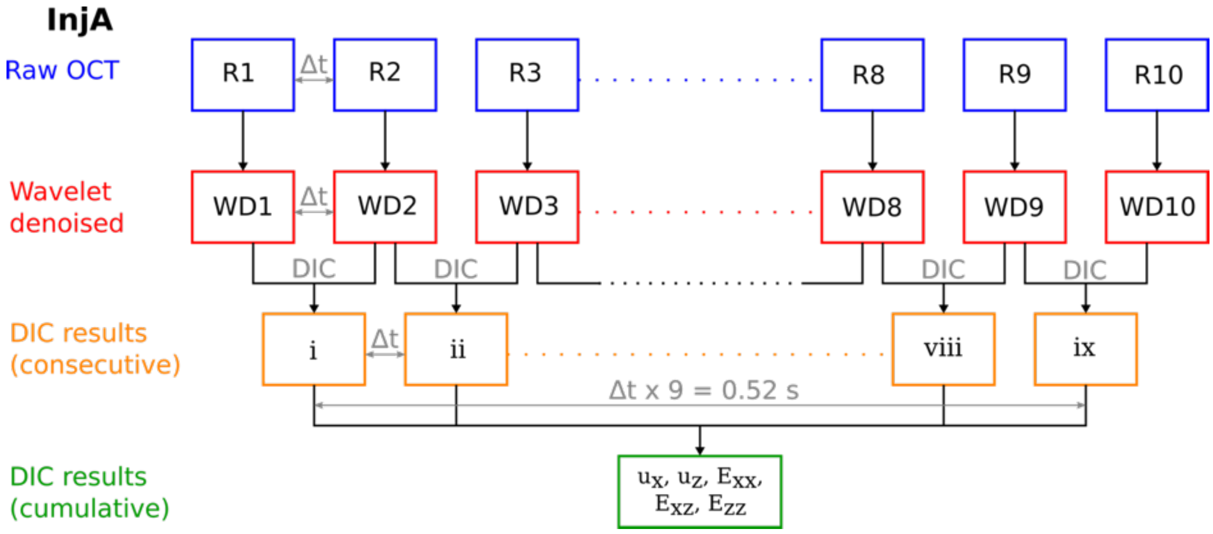

Fig. S4 | Time difference between DIC results for Inj1. Schematic for calculating the cumulative displacement and strain fields for Inj1 using DIC results from 9 pairs of wavelet denoised OCT images (Δt = 58 ms), with a time interval of 0.52 s between the first reference (WD1) and last denoised OCT image (WD10).

5.2 Inj2

The procedure for performing DIC on OCT images from Inj2, as shown in Fig. S5, was as follows:

- Averaging: Two consecutive raw OCT images (e.g. a and b) were averaged, (a+b)/2, using the technique described in Methods (image processing IV in Fig. S3). The time difference between consecutive averaged images, e.g. (a+b)/2 and (b+c)/2, was the same as that between the raw images (Δt = 13.5 ms).

- <u>DIC on consecutive images</u>: DIC was performed on consecutive averaged OCT images, e.g. (a+b)/2 and (b+c)/2, with the reference image being updated after each iteration. The time difference (Δt) between consecutive results was also 13.5 ms.
- <u>Cumulative DIC results</u>: The DIC results from 8 pairs of averaged images were combined in the NCorr software to provide u_x, u_z, E_{xx}, E_{xz} and E_{zz} with a total time difference of 108 ms between the first reference and last averaged OCT image.

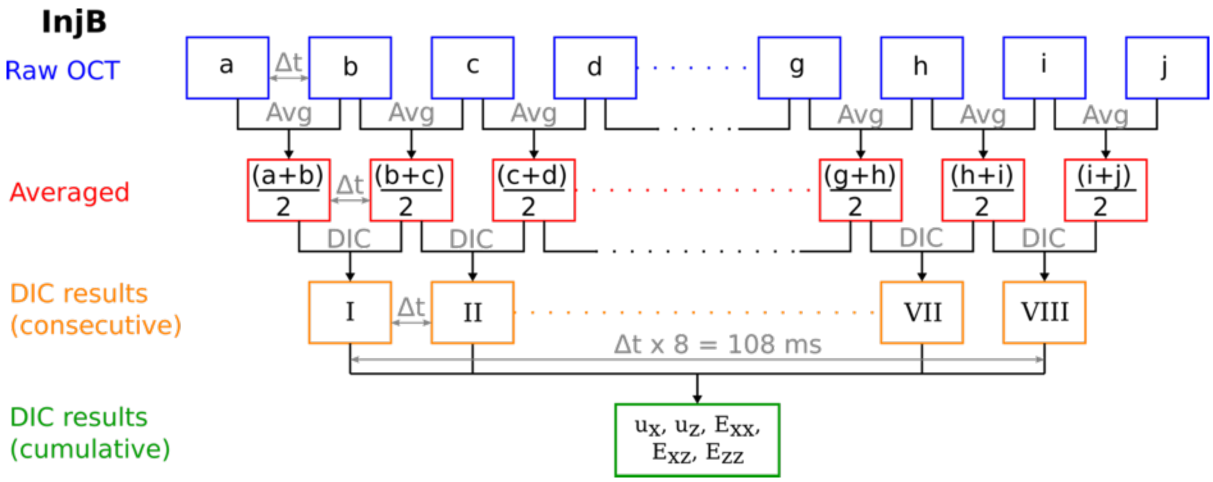

Fig. S5 | Time difference between DIC results for Inj2. Schematic for calculating the cumulative displacement and strain fields for Inj2 using DIC results from 8 pairs of averaged OCT images (Δt = 13.5 ms), with a time interval of 108 ms between the first reference, (a+b)/2, and last averaged OCT image, (i+j)/2.

6 Sample DIC data

Fig. S6 shows sample data for displacement fields u_x and u_z, and strain fields E_{xx}, E_{xz} and E_{zz} 1 s and 3.5 s after microneedle retraction. The 2D strain fields ε_{2D} are calculated based on equation (4) presented in the paper.

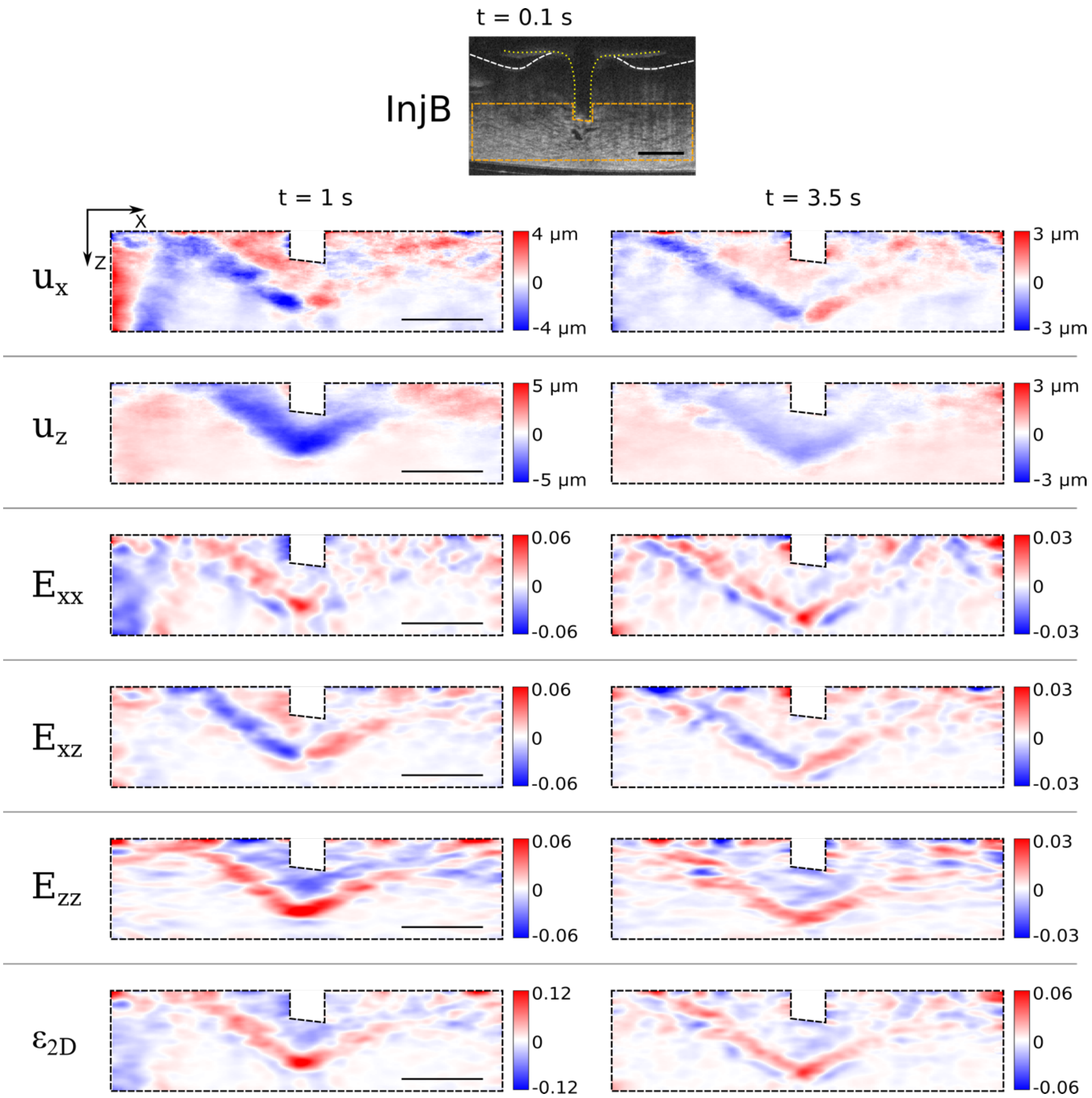

Fig. S6 | Sample DIC data. Displacement and strain fields for Inj2 (cumulative results for a time interval of 108 ms, considering 8 pairs of averaged OCT images). OCT image (top) shows ROI in orange dashed lines, MN in yellow dotted lines, and SC in white dashed lines. The displacement fields u_x and u_z, and strain fields E_{xx}, E_{xz}, and E_{zz} are calculated using DIC in Ncorr. The 2D strain fields ε_{2D} are calculated in MATLAB using equation (4) in the paper. The scale bars are 0.5 mm.

7 Strain fields before/after insertion and retraction

Fig. S7 shows strain fields and OCT images at different times during the injection.

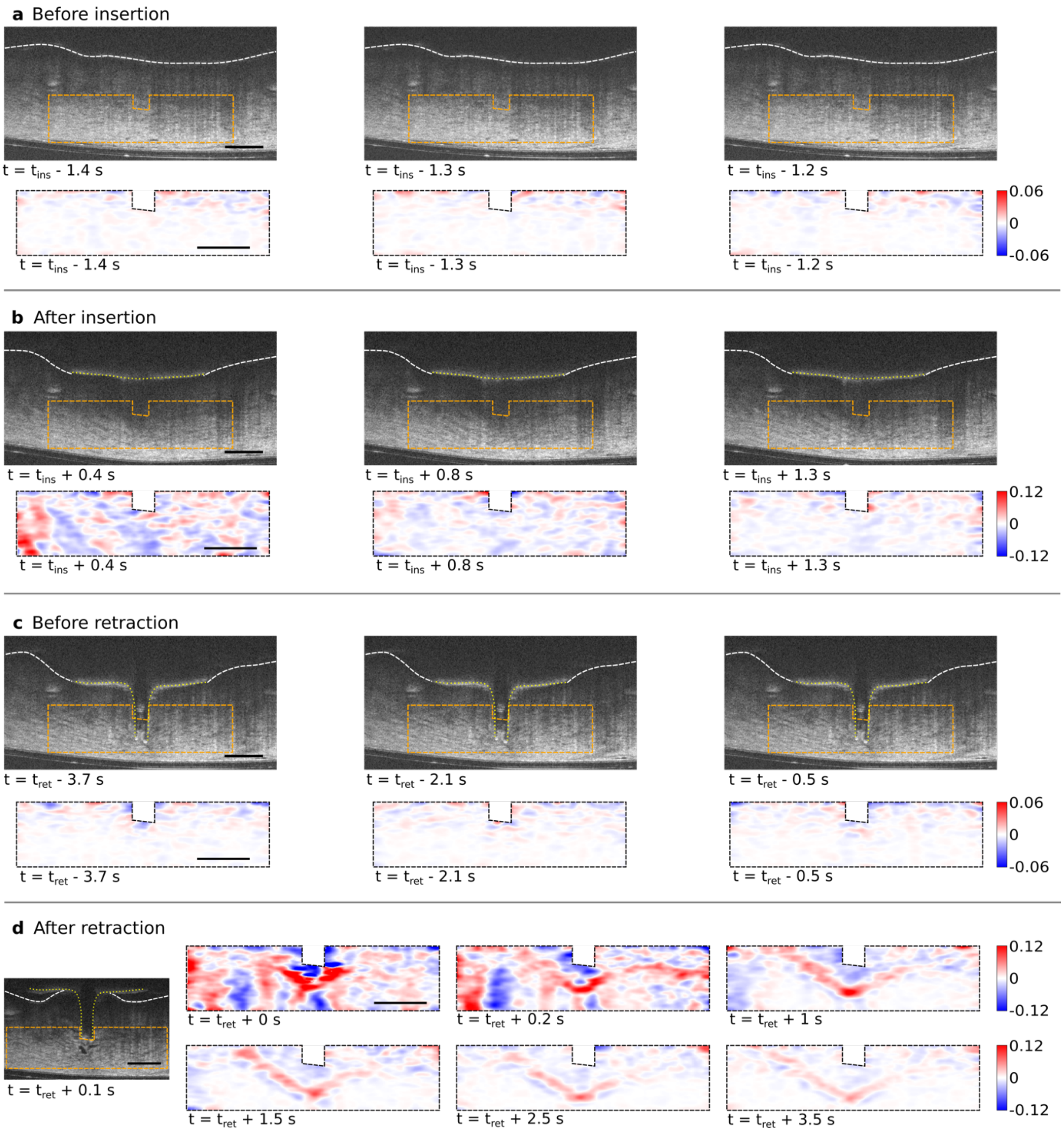

Fig. S7 | Strain maps at different times during the injection procedure. 2D strain maps (bottom) and corresponding OCT images at the last frame of DIC (top) before insertion (a), after insertion (b) and before retraction (c). d, Results after retraction shown in Fig. 4a in the manuscript. All scale bars are 0.5 mm.

Each strain map in Fig. R1 shows cumulative strain using DIC results on 8 pairs of consecutive (averaged) OCT images, with a total time difference between the reference OCT frame and the last OCT frame of 0.11 s. In the figure, t_{ins} denotes the time at which the microneedle is inserted into the skin, and t_{ret} denotes the time at which the microneedle completes retraction of 0.3 mm. The values of time are given in reference to (before or after) t_{ins} or t_{ret}, and the time stated below the strain results in Fig. R1 indicate the time for the last OCT frame used in the respective DIC

analysis. In each strain map, regions of local tissue expansion (positive strain) are shown in red, while regions of local tissue compression (negative strain) are shown in blue. The region of interest (ROI) for DIC is the same for all the results, and is shown using orange dashed lines in the OCT images and black dashed lines in the strain maps. The microneedle is shown using yellow dotted lines and the skin surface position is shown using white dashed lines.

The top panel in Fig. R1a shows OCT images for the experiment before microneedle insertion. Before insertion, as expected, the OCT images show no visible movement of the tissue and show some random generation of speckles due to noise that is more prominent towards the top of the OCT image. The corresponding strain maps, in the bottom panel of Fig. R1a, show regions of positive and negative strain, with the strain being larger near the top boundary of the ROI. This apparent strain could most likely be attributed to noise during OCT acquisition, which was more prominent in the upper parts of the OCT image due to the attenuation of signal through the skin. Additionally, the magnitude of limits for the strain maps before insertion [-0.06 0.06] are half of that for strain maps after insertion or retraction [-0.12 0.12] in Fig R1.

The frame rate of OCT acquisition (73.9 frames/s) was not high enough to capture the insertion process because the microneedle was inserted with an impact velocity of around 2 m/s. However, the deformation of the tissue after insertion could be captured by the OCT. Fig. R1b shows the OCT images and strain maps 0.4 s, 0.8 s and 1.3 s after insertion. For this particular experiment, the cross-sectional OCT image captured during insertion did not pass through the central axis of the microneedle and included only the base of the microneedle. Since, the required cross-section for imaging injections was usually adjusted after insertion, not all the OCT videos for insertion included the microneedle lumen. The strain maps for the cross-section shown in the top panel of Fig. R1b were calculated using the same region of interest for DIC as the other results. After insertion, the tissue below the microneedle is compressed, as indicated by the negative strain (blue) in the strain maps 0.4 s and 0.8 s after insertion. The high positive strain (red) at the left edge of the ROI 0.4 s after insertion could be due to the edges of the skin stretching as the central region of the tissue is compressed downwards by the microneedle.

Fig. R1c shows the OCT images of the last DIC frame (top) and corresponding 2D strain maps (bottom) 3.7 s, 2.1 s and 0.5 s before retraction. The limits of the strain maps are -0.06 to 0.06, which are half of that of Fig. R1d (since using the same scales resulted in the strain maps being predominantly white). Before retraction, the OCT images in Fig. R1c show no visible movement of the tissue. The strain maps show apparent strain as a result of noise, which is similar in magnitude to that before insertion (Fig. R1b). Fig. R1d shows the strain maps and sample OCT image after retraction of the microneedle, which were included in the manuscript in Fig 4a. A ‘V’ shaped region of high tissue expansion (positive strain) grows as the injection continues.

8 Strain map for initial injection

Fig. S8 shows an additional strain map for Fig. 4b presented in the paper. The strain map at 0.6 s has strain magnitude about an order of magnitude higher than that at 30.6 s. The ‘V’ shaped region of high tissue expansion (positive strain) widens from 0.6 s to 30.6 s, as shown in Fig. S8.

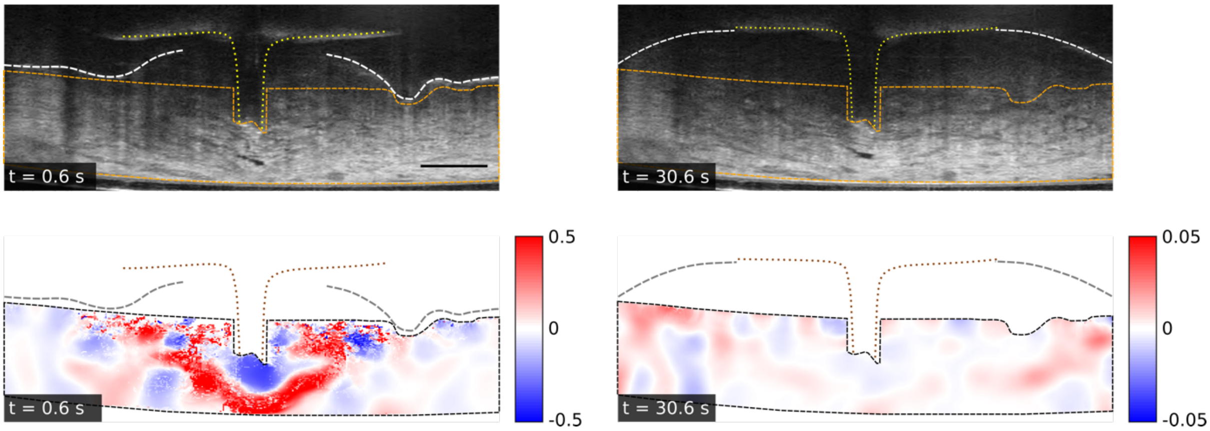

Fig. S8 | Strain map for initial injection. 2D maps of strain (ε_{2D}) for the region bounded by orange dashed lines in the OCT images. The position of the microneedle is shown using yellow dotted lines in OCT images and brown dotted lines in strain maps. The position of the skin surface is shown using white dashed lines in OCT images and grey dashed lines in the strain maps. Each strain maps shows cumulative strain using DIC results on 9 pairs of consecutive OCT images, with a total time difference between the reference OCT frame and the last OCT frame of 0.52 s. The rate of tissue expansion decreases over time, as shown by the decrease in strain magnitude from 0.6 s to 30.6 s. OCT images captured at 17.2 frames/s; P_{in} = 100 kPa and d_r = 0.3 mm. The scale bar is 0.5 mm

9 Correlation maps

We observed similar results for the growth of the expanded region for multiple injection experiments, as shown in Fig. S9 for 5 datasets. The shape of deforming tissue, indicated by dark pixels, was similar for all the injection tests. The first four sets (InjA – InjD) are for injection of water into tissue and a retraction distance of 0.3 mm, the last set (InjE) is for injection of viscous fluid (65 °Bx sugar solution) and a retraction distance of 0.2 mm. The amount of deforming tissue reduces from 1 s to 5 s for all injections. Note: InjA and InjB are the same as Inj1 and Inj2 respectively.

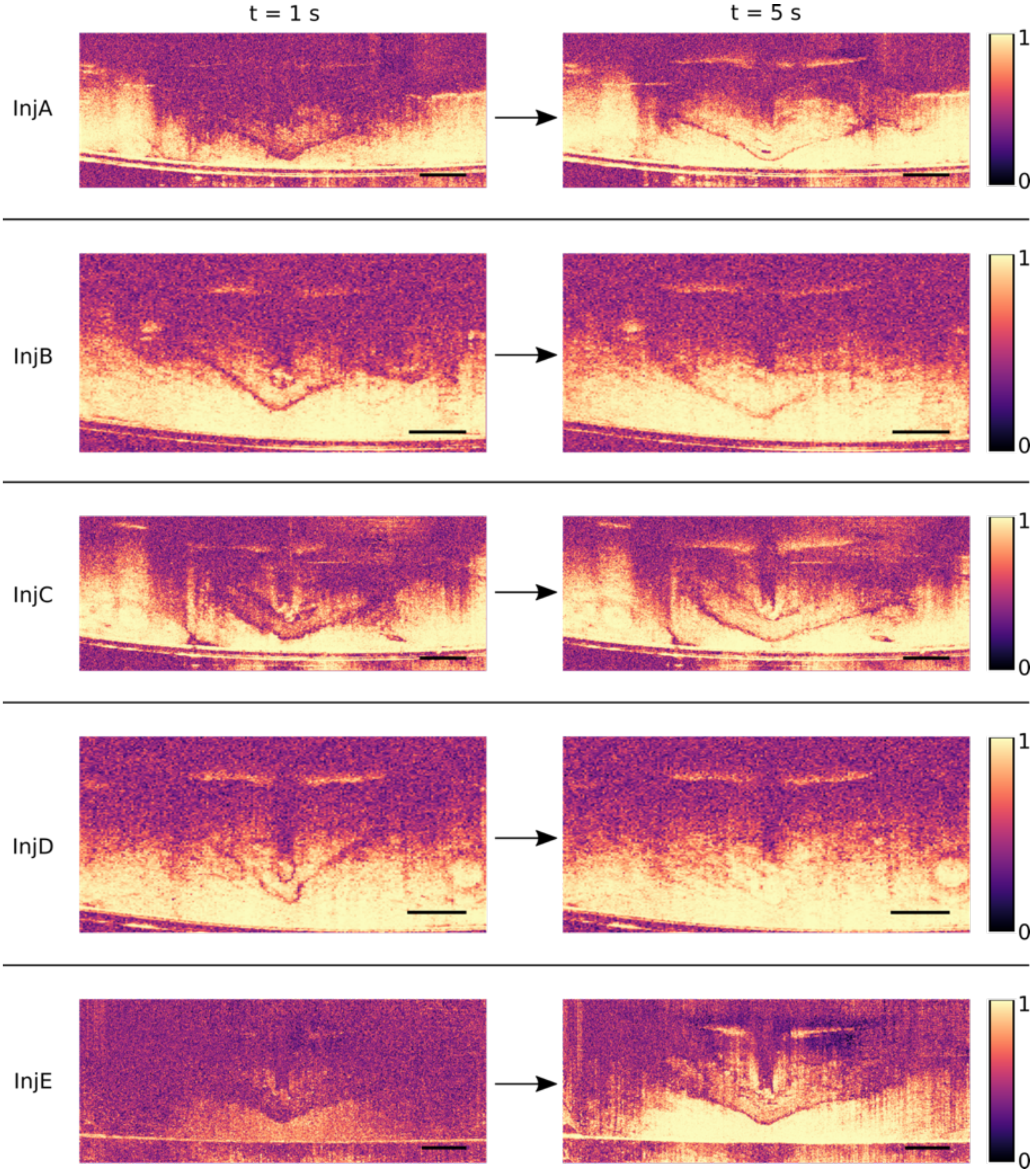

Fig. S9 | Correlation maps of other injections. Correlation maps for 5 sets of injection experiments (at 1 s and 5 s), showing 'V' shaped bands of low correlation (dark pixels) indicating movement of tissue. Bright pixels indicate stationary tissue. P_{in} = 100 kPa; d_r = 0.3 mm (InjA – InjD) and 0.2 mm (InjE); Injected fluid = water (Inj A – InjD), and 65°Bx sugar solution (InjE). The black scale bars are 0.5 mm.

10 Continuous retraction

Similar to multiple retractions, slow and continuous retraction of the microneedle resulted in higher injection volume as compared to a single step retraction. Fig. S10 shows the correlation maps and pictures of the tissue holder for an injection with continuous retraction of the microneedle. The microneedle is slowly retracted at a speed of 0.1 mm/s up to a distance of 1 mm. The first two time frames shown are during retraction, while the last two are after the completion of retraction (1 mm). The injected fluid, water, is mixed with green dye for demonstrating the uptake of fluid from the outside. Green dye was not used for the other experiments described in the paper. The injection pressure was set to 100 kPa.

The continuous retraction of 1 mm would not have been possible with a single step retraction, since the height of the microneedle used was 0.7 mm. Like the successive retractions described in the paper, the continuous retraction allows the tissue to swell as the microneedle is retracted, prolonging the microinjection mode of flow and resulting in a greater flowrate (and volume) than a single retraction case. The swelling of the stratum corneum and uptake of water, as seen in Fig. S10 and Supplementary video 7, results in the formation of a raised papule.

The correlation maps show the regions of deforming and stationary tissue. The first two correlation maps for 2 s and 6 s can be compared with those for the other injections (InjA – InjE). As shown earlier in Fig. S9, for the other injections (InjA – InjE) with step retraction of the microneedle (0.2 mm – 0.3 mm), the region of deforming tissue decreased from 1 s to 5 s, forming a thin band of deforming tissue (‘V’ shaped) at 5 s. However, for the continuous retraction case (InjF), the region of deforming tissue increases from 2 s to 6 s, rather than decreasing, because at 6 s the microneedle is still retracting – the retracting microneedle allows the skin to continuously expand and absorb fluid, rather than acting as a barrier for tissue expansion (like in the step retraction case).

The narrow band of deforming tissue, separating the expanded tissue and stationary tissue, can be seen at 12 s and 16 s in Fig. S10. The top part of the correlation maps showing low correlation coefficients can be attributed to noise because in the top half of the image, the OCT signal was almost completely attenuated. The region of expanded tissue is much larger in this case, covering the skin sample almost completely, unlike (for instance) for the step retraction case for InjA (Fig. S9), where at 5 s the region of expanded tissue was bounded by the ‘V’ shape.

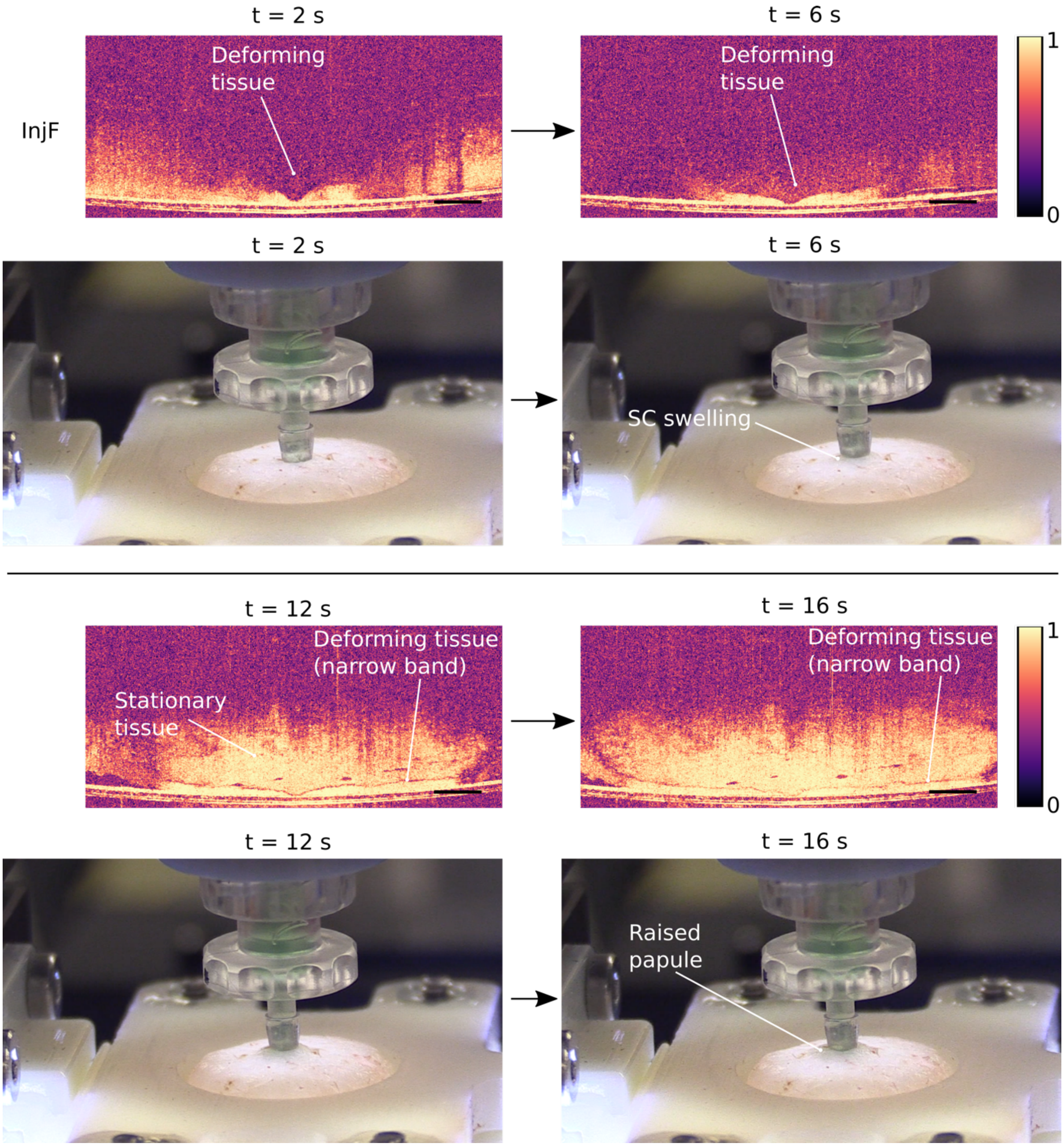

Fig. S10 | Continuous retraction of the microneedle. Correlation maps and images at different times during the injection of water (mixed with green dye) into skin. The microneedle is retracted continuously from 0 s to 10 s to a distance of 1 mm. The black scale bars are 0.5 mm.

11 Supplementary tables

Table S1 shows the sensors and actuators used in the experimental setup used for the work reported in the paper. Table S2 indicates the settings of the OCT system for the two frame rates reported in the paper.

Table S1: Sensors and actuators used in the experimental setup and the corresponding parameters controlled or measured.

Controlled Quantity	Symbol	Actuator	Range
Input Pressure	$P_{in,t}$	Elveflow OB1 Pressure Controller	0 – 2 bar (0 – 200 kPa)
Retraction distance	d_r	Zaber T-LSM050A Motorized Stage	50.8 mm (0.00022 mm/s – 7 mm/s)

Measured Quantity	Symbol	Sensor	Range
Input Pressure	$P_{in,m}$	Elveflow OB1 Pressure Controller	0 – 2 bar (0 – 200 kPa)
Flowrate	Q	Elveflow Microfluidic Flow Sensor	0 – 1 ml/min
Pressure	P_m	Elveflow Pressure Sensor	0 – 30 psi (0 - 206.843 kPa)

Table S2: The scanning parameters used for OCT acquisition at the two frame rates used in the paper.

Parameter	Inj1	Inj2
Frame rate (frames/s)	17.2	73.9
Frame acquisition time (s)	0.0581	0.0135
Acquisition size Z (pixels)	1024	1022
Acquisition size Z (mm)	3.61	3.60
Acquisition size X (pixels)	1274	700
Acquisition size X (mm)	4.5	4
Pixel spacing in Z (mm/pixel)	0.0035	0.0035
Pixel spacing in X (mm/pixel)	0.0035	0.0057
A scan averaging	2	1
B scan averaging	1	1
